# Shared splicing dysregulation in heart failure associated with dilated and ischaemic cardiomyopathy and spatial specificity across cardiac regions

**DOI:** 10.1093/cvr/cvag068

**Published:** 2026-03-24

**Authors:** Marta Furtado, Pedro Barbosa, Ana Wemans, Lu Zhang, Andrew Lumley, Teresa Carvalho, Patrícia Napoleão, Przemyslaw Leszek, Maria Carmo-Fonseca, Yvan Devaux, Sandra Martins

**Affiliations:** Gulbenkian Institute for Molecular Medicine, Pólo de Oeiras, Rua Quinta Grande, 6, 2780-156 Oeiras, Portugal; Faculdade de Medicina da Universidade de Lisboa, Avenida Professor Egas Moniz, 1649-028 Lisboa, Portugal; Gulbenkian Institute for Molecular Medicine, Pólo de Oeiras, Rua Quinta Grande, 6, 2780-156 Oeiras, Portugal; Gulbenkian Institute for Molecular Medicine, Pólo de Oeiras, Rua Quinta Grande, 6, 2780-156 Oeiras, Portugal; Bioinformatics & AI Unit, Luxembourg Institute of Health, Strassen, Luxembourg; Cardiovascular Research Unit, Department of Precision Health, Luxembourg Institute of Health, Strassen, Luxembourg; Faculdade de Medicina da Universidade de Lisboa, Avenida Professor Egas Moniz, 1649-028 Lisboa, Portugal; Gulbenkian Institute for Molecular Medicine, Pólo de Oeiras, Rua Quinta Grande, 6, 2780-156 Oeiras, Portugal; The Heart Failure and Transplantology Department, Institute of Cardiology, Warsaw, Poland; Gulbenkian Institute for Molecular Medicine, Pólo de Oeiras, Rua Quinta Grande, 6, 2780-156 Oeiras, Portugal; Faculdade de Medicina da Universidade de Lisboa, Avenida Professor Egas Moniz, 1649-028 Lisboa, Portugal; Cardiovascular Research Unit, Department of Precision Health, Luxembourg Institute of Health, Strassen, Luxembourg; Faculdade de Medicina da Universidade de Lisboa, Avenida Professor Egas Moniz, 1649-028 Lisboa, Portugal

**Keywords:** dilated cardiomyopathy, ischaemic cardiomyopathy, alternative splicing, heart failure

## Abstract

**Aims:**

Alternative splicing plays a critical role in cardiac development and function and becomes dysregulated in heart failure. Although splicing defects have been described in both dilated (DCM) and ischaemic (ICM) cardiomyopathy, the extent to which these alterations contribute to disease mechanisms and how they are spatially distributed across cardiac regions remains poorly understood. This study aimed to profile alternative splicing events across the left ventricle (LV), right ventricle (RV), and interventricular septum (IVS) in end-stage heart failure patients with DCM and ICM, and to investigate potential regulatory factors driving these changes.

**Methods and results:**

RNA-seq was performed on LV tissue from patients with DCM (*n* = 10), ICM (*n* = 11), and non-failing controls (*n* = 5), and analysed using three complementary splicing tools to maximize event detection. This integrative approach consistently revealed widespread splicing alterations in heart failure samples compared to controls, with substantial overlap between DCM and ICM. Motif enrichment analysis implicated the RNA-binding protein QKI as a potential splicing regulator in heart failure. Validation of six selected splicing events by qRT–PCR in a larger cohort (54 DCM, 45 ICM, 23 controls) confirmed shared dysregulation in DCM and ICM. While splicing alterations in *CAMK2D* and *PDLIM3* were detected across the LV, RV, and IVS in both DCM and ICM, other transcripts (*MYL6*, *ESRRG*, *EYA4*, and *SORBS1*) differed between DCM and ICM, with DCM showing broader chamber-wide splicing alterations.

**Conclusion:**

This study presents the first multi-chamber analysis of splicing in human heart failure, revealing a set of splicing events commonly dysregulated in DCM and ICM. These findings support the notion that splicing dysregulation can be a shared molecular response to advanced cardiac remodelling, rather than a driver of aetiology-specific pathology. We further uncovered distinct spatial patterns: in DCM, splicing alterations were consistently observed across all cardiac chambers, likely reflecting diffuse myocardial involvement. In contrast, certain splicing changes in ICM were restricted to the LV, consistent with the focal nature of ischaemic injury.

**Time of primary review: 43 days**

**See the editorial comment for this article ‘RNA splicing in human cardiomyopathies: shared landscape in diverse pathogenic paths’, by Y. Wang**  ***et al*****., https://doi.org/10.1093/cvr/cvag070.**

## 1. Introduction

Heart failure (HF) is a growing global public health and economic challenge, especially in ageing societies. Despite advances in pharmacological treatments, device-based therapies, and diagnostic approaches, HF remains the leading cause of hospitalization in individuals over the age of 65, and mortality rates remain unacceptably high.^1^ HF with reduced ejection fraction (HFrEF), marked by a substantial decline in systolic function, frequently represents the final stage in the progression of various cardiac diseases. Two of the most prevalent causes are ischaemic cardiomyopathy (ICM), primarily resulting from coronary artery disease,^2^ and dilated cardiomyopathy (DCM), characterized by left ventricular enlargement and impaired contractility.^3^ Although DCM can be triggered by non-genetic factors such as hypertension, valve disease, inflammation, and toxins, up to 40% of cases have a genetic basis,^4^ involving pathogenic variants across more than ten functional gene categories.^5^

Transcriptomic studies have aimed to uncover molecular signatures associated with end-stage HF. Bulk RNA sequencing (RNA-seq) has shown both shared and disease-specific aetiologies,^6^ while single-nucleus RNA-seq has revealed similar expression patterns across DCM, ICM, and hypertrophic cardiomyopathy.^7,8^ These findings suggest that, despite differing aetiologies, cardiomyopathies converge on a common terminal gene expression programme.

In addition to transcriptional regulation, RNA splicing plays a critical role in gene expression. The removal of introns and ligation of exons from pre-mRNA enables the generation of mature mRNAs, often through alternative splicing (AS), which expands protein diversity. AS can result in exon skipping, inclusion of mutually exclusive exons, alternative 5′ or 3′ splice site usage, and intron retention. The resulting transcript isoforms can differ in localization, function, or stability.^9,10^ Splicing dysregulation is implicated in numerous human diseases, including inherited and acquired forms of heart disease.^9,10^

A pioneer genome-wide splicing analysis in ICM patients identified abnormally spliced mRNAs encoding proteins involved in cardiac structure and function.^11^ A subsequent deep RNA-seq study of 97 DCM hearts and 108 non-failing (NF) controls identified differential splicing of 1212 exons in 899 genes.^12^ However, whether these alterations represent disease-driving events or secondary consequences of pathological remodelling remains unresolved. Comparative analysis of splicing changes across different cardiomyopathy subtypes may help clarify this issue. Kong and colleagues reported similar splicing alterations in mRNAs encoding four sarcomere proteins in both ICM and DCM samples.^11^ Still, comprehensive genome-wide comparisons of splicing profiles between ICM and DCM are lacking.

**Figure 1:**
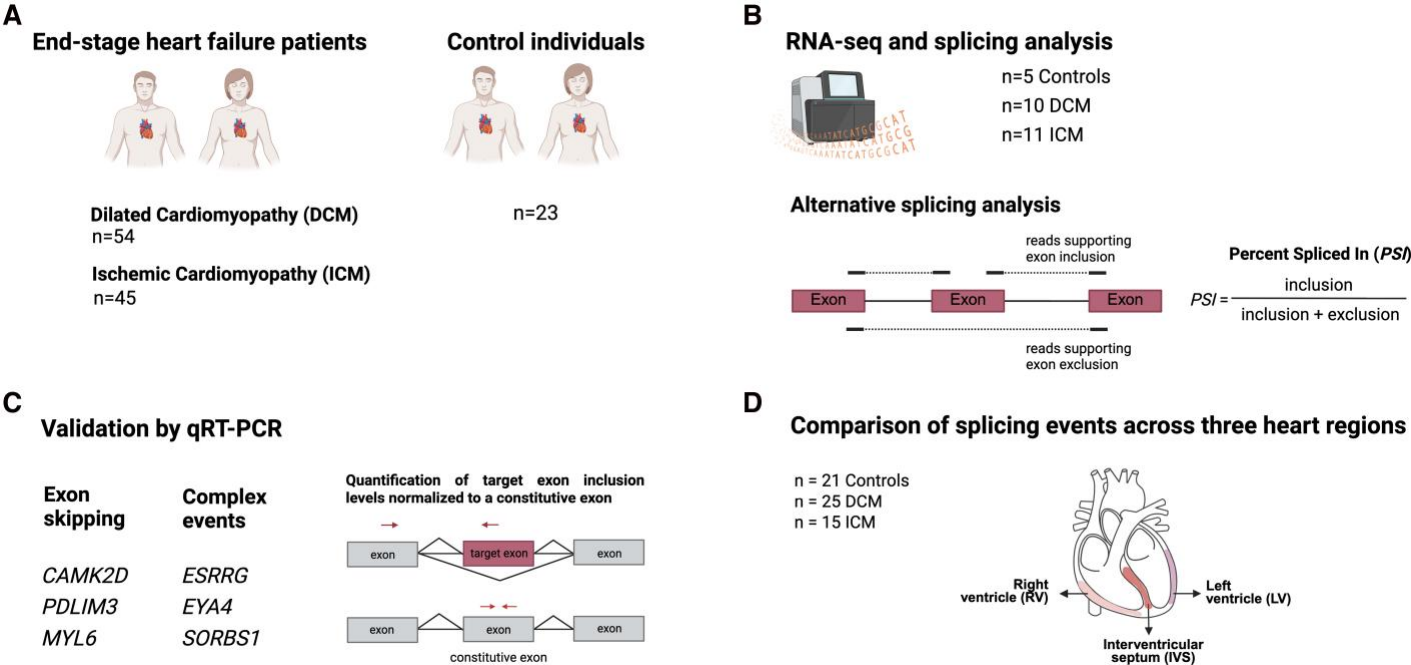
Study design. (*A*) Cardiac samples were collected from 54 end-stage heart failure patients with dilated cardiomyopathy (DCM) and 45 patients with ischaemic cardiomyopathy (ICM), along with 23 samples from non-failing control hearts. (*B*) RNA-seq data was generated from the left ventricle of 10 DCM, 11 ICM patients and 5 controls. The formula used to calculate the Percent Spliced In (PSI) value is indicated. (*C*) Selected splicing events in the indicated genes were quantified by qRT-PCR in all available samples. Primer pairs (arrows) were designed to amplify transcripts that include or skip the differentially spliced target exon. Expression levels were normalized to a constitutive exon present in all transcript isoforms of the gene. (*D*) Dysregulated splicing events detected in the left ventricle (LV) were analysed by qRT-PCR in matched samples from the right ventricle (RV) and interventricular septum (IVS).

In this study (*Figure 1*), we analysed RNA-seq data from LV tissue of end-stage HF patients with DCM (*n* = 10) and ICM (*n* = 11), as well as NF controls (*n* = 5). We identified widespread splicing dysregulation in HF compared to controls, with substantial overlap between DCM and ICM. Validation of selected splicing events by qRT–PCR in an expanded cohort (DCM, *n* = 54; ICM, *n* = 45; controls, *n* = 23) confirmed shared patterns of dysregulation in the left ventricle (LV) across both forms of cardiomyopathy. Notably, some splicing alterations such as those in *CAMK2D* and *PDLIM3* were consistently detected across the LV, right ventricle (RV), and interventricular septum (IVS), suggesting regulation by broadly activated disease pathways. In contrast, other events such as those in *MYL6*, *ESRRG*, *EYA4*, and *SORBS1* exhibited more spatially restricted patterns, particularly in ICM. These differences may reflect localized ischaemic injury in ICM vs.more diffuse disruption in DCM.

## 2. Methods

### 2.1 Human samples

In this study, we analysed human heart samples from 54 DCM patients, 45 ICM patients and 23 controls. The diagnosis of ICM or DCM was based on patient's history, echocardiography, coronary angiography, and additional relevant data. Patient samples were obtained from explanted failing hearts unsuitable for transplantation due to a variety of reasons other than HF (hepatitis B, cytomegalovirus infection, extensive damage during harvesting, and/or donor/recipient size mismatch). Tissue samples were collected at the time of heart transplant. Control heart samples were collected *post-mortem* from individuals with either head injury (*n* = 8) or subarachnoid haemorrhage (*n* = 15). Tissue samples were collected from LV, RV, and IVS. The protocol has been approved by the Local Ethics Committee (number IK-NP-0021-48/846/13; 09 April 2013, and IK-NPIA-0021-34/1699/18, 15 May 2018). The investigation conformed to the principles outlined in the Declaration of Helsinki. All patients participating provided written informed consent for the study protocol after a detailed explanation of the principles prior to inclusion into the study. Neither donors nor their relatives completed National Refusal List. Samples were snap frozen and stored at −80°C until analysis. Clinical and demographic information of study participants is given in Supplementary material online, *Table S1A* and *B*.

### 2.2 RNA sequencing (RNA-Seq)

Total RNA isolation and purification from heart samples was performed as previously described in Firat *et al.*^13^ (see Supplementary Methods). Sequencing libraries were prepared with the Illumina TruSeq^TM^ stranded mRNA and total RNA kit and were sequenced on an Illumina NextSeq 500 platform (see Supplementary Methods for details).

### 2.3 Splicing analysis

To reliably capture global splicing changes across DCM, ICM, and control datasets, we used three well-established and complementary methods: *rMATS* v4.1.2, *MAJIQ* v2.5.1, and *vast-tools* v2.5.1. For differential splicing analysis, we considered events with absolute dPSI ≥ 0.1 between the two conditions. We provide a detailed description in Supplementary Methods.

### 2.4 Quantitative RT–PCR

We performed cDNA synthesis, RT, and qRT–PCR as described in Supplementary Methods. All RT and qRT–PCR primers are shown in Supplementary material online, *Table S2A* and *S2B*.

## 3. Results

### 3.1 Cross-tool analysis reveals shared splicing dysregulation in dilated and ischaemic cardiomyopathy

Total RNA was extracted from left ventricular samples of 10 patients with DCM, 11 with ICM and 5 non-failing controls, and subjected to deep RNA sequencing (*Figure 1B*). On average, 210 ± 81 million mapped paired-end reads were generated per sample, providing sufficient depth for a detailed characterization of gene expression and RNA splicing.

Accurate detection of AS events from RNA-seq data is methodologically challenging. Although numerous tools have been developed for splicing analysis, benchmarking studies have shown that these tools often yield divergent results, with many events detected exclusively by a single method.^14–16^ To overcome the limitations of relying on a single analysis pipeline, we employed an integrative approach using three event-based splicing tools: *rMATS*,^17^  *vast-tools*^18^ and *MAJIQ*.^19^

All three tools detect and quantify AS events using the Percent Spliced in (PSI) metric, which estimates the proportion of transcripts that include a given event (e.g. an exon, retained intron, or alternative splice junction) relative to the total number of transcripts for that gene. While conceptually similar, these tools differ in how they define, detect, and statistically evaluate splicing events, providing complementary strengths.

*rMATS* excels at detecting and quantifying common types of AS events, including exon skipping, alternative 5ʹ and 3ʹ splice sites, mutually exclusive exons, and intron retention, by comparing splicing patterns between sample groups using a statistical model that accounts for biological variability.^17,20^

*vast-tools* is particularly well suited for the sensitive detection of conserved splicing events across diverse cell types and disease conditions. It leverages a highly curated database of annotated AS events, enabling high-confidence quantification.^18^

*MAJIQ* is distinctively effective at capturing complex or non-canonical splicing events, such as multiple alternative junctions within a single region, which may be missed by other exon-centric approaches.^19^

**Figure 2:**
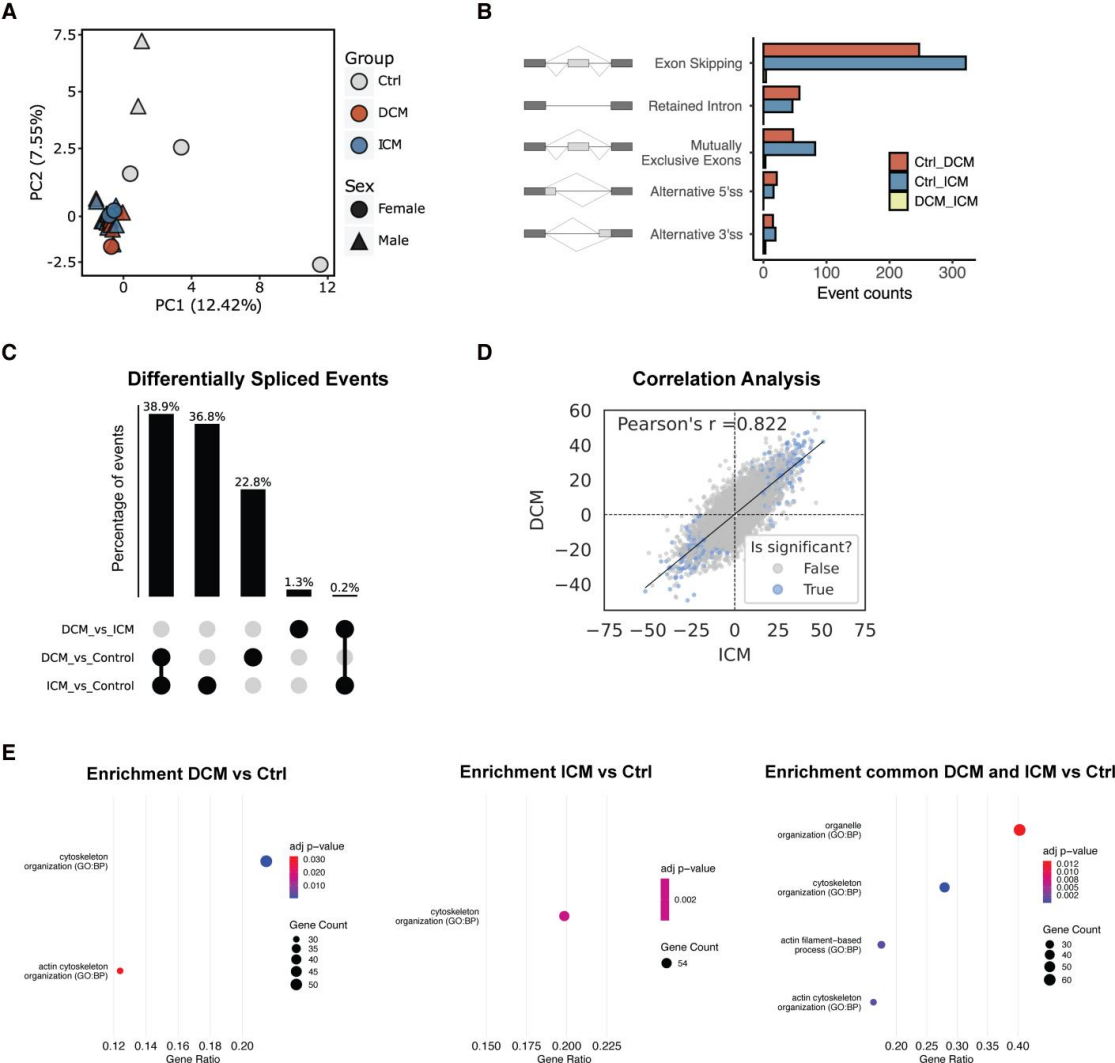
RNA-seq analysis using *rMATS* identifies shared splicing changes in DCM and ICM hearts. (*A*) Principal component analysis (PCA) based on PSI values demonstrates clustering of DCM and ICM splicing profiles, distinct from controls (*n* = 10 DCM, *n* = 11 ICM and *n* = 5 controls). (*B*) Distribution and types of events identified as differentially spliced in pairwise comparisons between controls and diseased groups (DCM and ICM). Differential splicing events were defined using a false discovery rate (FDR) < 0.05 and an absolute change in Percent Spliced In (ΔPSI) > 0.1. (*C*) UpSet plot showing the percentage of differentially spliced events identified in pairwise comparisons: DCM vs. Control, ICM vs. Control, and DCM vs. ICM, as well as the subset of events shared between DCM and ICM when compared to Control. (*D*) Pearson's correlation analysis of ΔPSI values between DCM and ICM relative to controls, indicating a high degree of overlap in splicing changes. (*E*) Pathway enrichment analysis of genes containing differentially spliced events identified in DCM and ICM compared to controls, as well as in the subset of events shared between both cardiomyopathy subtypes.

To evaluate global differences in splicing patterns between sample groups, we performed principal component analysis (PCA) using the PSI values obtained independently from each of the three splicing tools. The three tools revealed a consistent pattern: DCM and ICM samples clustered closely together, indicating a high degree of similarity in splicing profiles across these two forms of heart failure (*Figure 2A* and Supplementary material online, *Figures S1A* and *S2A*). In contrast, control samples were more heterogeneous and clearly separated from the heart failure samples.

Pairwise comparisons among the three groups using all three tools consistently demonstrated that most splicing differences occurred between failing hearts and controls, whereas only a limited number of events distinguished DCM from ICM samples (see Supplementary material online, *Table S3*).

Differential splicing analysis using *rMATS* identified 639 differentially spliced events, with exon skipping being the most frequently observed splicing alteration (*Figure 2B*). Similarly, *vast-tools* detected 340 differentially spliced events, again with a predominance of exon skipping events (see Supplementary material online, *Figure S1B*). In contrast, *MAJIQ*, which is specifically designed to capture a broader spectrum of splicing alterations, identified a total of 1599 differentially spliced events. These included exon skipping, but also a substantial number of alternative first and last exons, as well as intron retention events (see Supplementary material online, *Figure S2B*).

The variation in the number and types of splicing events detected by each tool reflects their distinct analytical strategies and sensitivities, reinforcing the value of a multi-tool approach to comprehensively characterize splicing dysregulation in heart failure. Consistent with previous benchmarking studies,^14–16^ we observed limited overlap between tools: only a small proportion of events were detected by more than one method, and just seven events were consistently identified by all three tools (see Supplementary material online, *Table S4*).

As a next step to assess cross-tool agreement, we examined whether the same genes were identified as differentially spliced, regardless of the specific splicing event. For this analysis, we considered all splicing events detectable by each tool. We identified 35 genes reported as differentially spliced by all three tools, even though the precise splicing events often differed between methods (see Supplementary material online, *Table S4*). Notably, this set includes genes with well-established roles in cardiac function such as those encoding titin (*TTN*), LIM domain only 7 (*LMO7*), and estrogen-related receptor gamma (*ESRRG*), supporting the idea that these genes undergo altered splicing regulation in heart failure. The divergence in the specific events detected likely reflects the presence of multiple, distinct splicing changes within the same gene. For example, one tool may detect exon skipping, while another captures alternative first or last exons or intron retention.

Remarkably, all three tools consistently revealed that 36–50% of the differentially spliced events identified in comparisons between patients and controls were shared between DCM and ICM, with minimal differential splicing between DCM and ICM hearts (*Figure 2C* and Supplementary material online, *Figures S1C* and *S2C*). Using *rMATS*, we found that 38.9% of the differentially spliced events identified in pairwise comparisons between HF samples and controls were shared between DCM and ICM. In contrast, 36.8% of the events were uniquely detected in the ICM vs. control comparison, and 22.8% were uniquely detected in the DCM vs. control comparison. Notably, only 1.3% of splicing events differed between DCM and ICM hearts.

A strong positive correlation (*r* ≈ 0.8) between DCM and ICM ΔPSI values relative to controls (*Figure 2D* and Supplementary material online, *Figures S1D* and *S2D*) further indicates that splicing alterations occur in a similar direction across both heart failure types (i.e. exons that show increased inclusion in DCM also tend to exhibit increased inclusion in ICM).

Enrichment analysis revealed that genes undergoing AS, as identified by *rMATS* and *MAJIQ*, were consistently enriched in biological pathways related to cytoskeleton organization and actin filament-based processes (*Figure 1E* and Supplementary material online, *Figure S2E*). No significant pathway enrichment was observed for genes identified as differentially spliced using *vast-tools*. Considering the small percentage of genes (0–6%) found to be differentially spliced between DCM and ICM (see Supplementary material online, *Table S3*), no enrichment was observed for specific pathways, suggesting that the detected differences may be functionally insignificant or reflect random variability.

### 3.2 Exploring mechanisms of splicing dysregulation in heart failure: RNA-binding protein expression and binding motif analysis

Splicing is regulated through multiple mechanisms, including the action of RNA-binding proteins (RBPs) that recognize specific sequence elements in the pre-mRNA known as splicing enhancers and silencers.^9^ In the heart, a variety of splicing regulatory RBPs are expressed, playing a crucial role in generating diverse mRNA isoforms essential for normal cardiac development and function.^10^ Disruptions in the activity of these proteins can have significant pathological consequences. Therefore, we investigated whether splicing factor mRNA levels were altered in our datasets.

**Figure 3:**
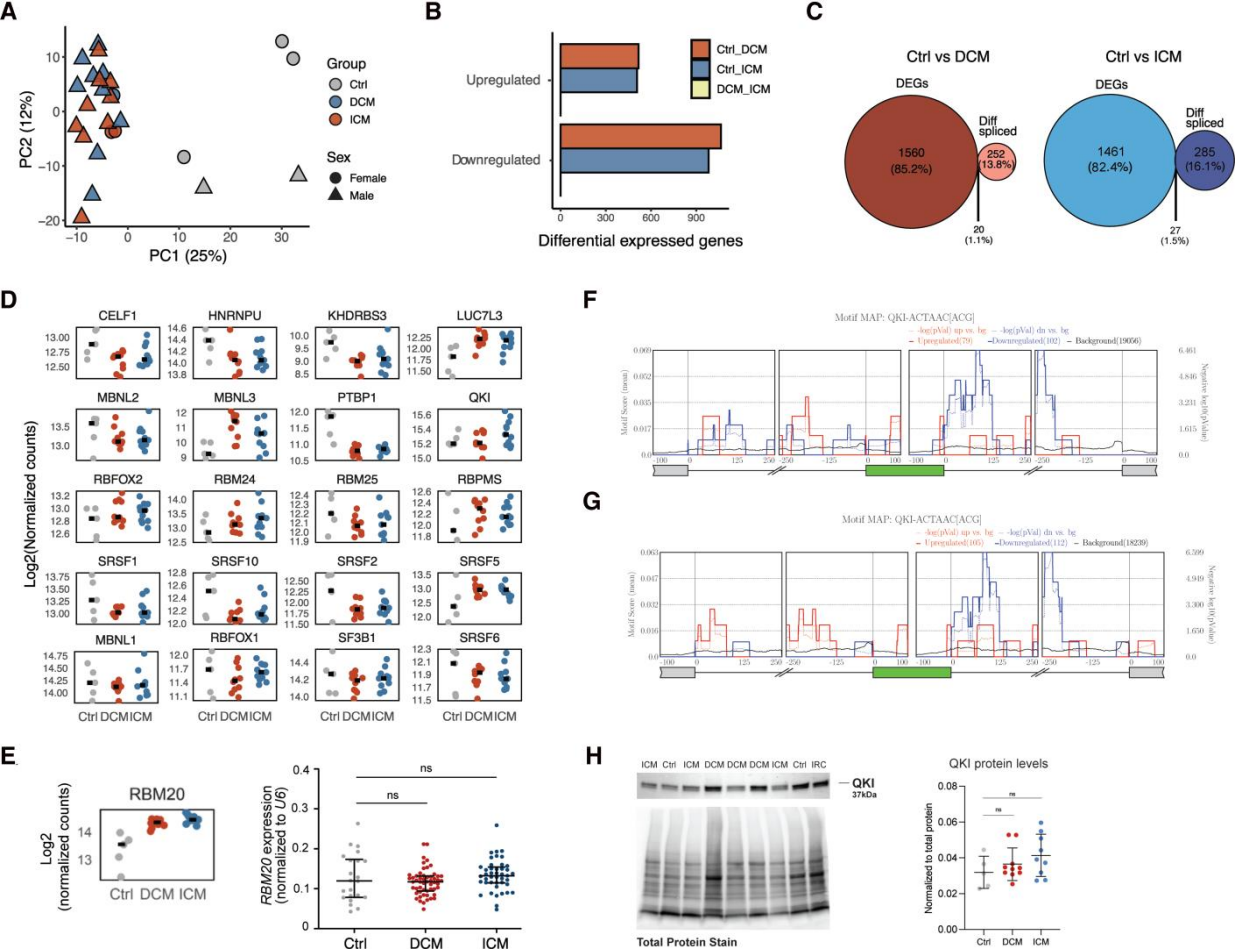
Gene expression dysregulation in heart failure. (*A*) Principal Component Analysis (PCA) based on rlog-transformed count data. Analysis was performed using the 1000 genes with the highest variance across samples. (*B*) Number of differentially expressed genes identified for each pairwise comparison between controls, DCM, and ICM samples. (*C*) Venn diagram illustrating the overlap between differentially expressed genes and genes containing at least one significant splicing event, as detected by *rMATS*. (*D*) Normalized gene expression (log2) of a curated list of cardiac-associated splicing factors. (*E*) Log-transformed, normalized expression values of *RBM20* calculated using DESeq2's median of ratios method and levels of *RBM20* mRNA relative to *U6* snRNA measured by qRT-PCR. Statistical analysis was performed using Brown-Forsythe and Welch ANOVA tests, followed by Games-Howell's multiple comparisons test. Differences were not statistically significant, *ns* (*P* > 0.05). (*F*,G) RNA-binding protein enrichment analysis using *rMAPS2* for differentially spliced events in DCM (*F*) and ICM (*G*) for QKI. (*H*) Western blot analysis of QKI protein levels in human heart tissue. Representative Western blot showing QKI protein abundance in a subset of control, DCM, and ICM heart biopsies (top). Total protein staining is shown below. Full image of QKI staining is presented in Supplementary material online, *Figure S3G*, Membrane 2. The graph on the right shows quantification of QKI protein levels normalized to total protein across the full cohort (controls, *n* = 5; DCM, *n* = 10; ICM, *n* = 9) (see Supplementary material online, *Figure S3G*, Membranes 1–3). Statistical analysis was performed using ordinary one-way ANOVA test followed by Dunnett's multiple comparisons test. Differences were not statistically significant, ns (*P* > 0.05).

First, we examined global differential gene expression in DCM, ICM, and control groups. PCA revealed clustering of DCM and ICM samples, clearly separating them from non-diseased controls (*Figure 3A*). Notably, no genes were found to be differentially expressed between DCM and ICM cases (*Figure 3B*). Most differentially expressed genes were downregulated in DCM and ICM samples compared to controls (*Figure 3B* and Supplementary material online, *Table S5*). This widespread reduction in mRNA levels led us to hypothesize that disrupted splicing might contribute to the generation of unproductive transcripts, triggering their degradation through nonsense-mediated decay.^21^ However, we found that only <1.5% of genes were at the same time differentially spliced and differentially expressed in DCM and ICM samples (*Figure 3C* and Supplementary material online, *Figures S3A* and *S3B*), suggesting that splicing alterations are unlikely to be the primary driver of global mRNA downregulation in heart failure. Our analysis further revealed that the number of differentially expressed genes exceeds the number of differentially spliced genes, suggesting that transcriptional dysregulation is more pervasive than splicing alterations in heart failure. Pathway enrichment analysis of the differentially expressed genes in DCM and ICM revealed enrichment profiles distinct from those observed for genes undergoing splicing alterations. While differentially spliced genes were primarily associated with pathways related to cytoskeleton organization, the top pathways enriched for differentially expressed genes were linked to immune function and inflammatory responses (see Supplementary material online, *Figure S3C*, *D* and *E*). These results further support our observation that most differentially spliced genes are not differentially expressed, reinforcing that distinct functional gene categories are regulated at the levels of expression and splicing.

We next focused on the expression of RBPs with known roles in splicing regulation. To this end, we examined a list of splicing-associated RBPs annotated by the ENCODE consortium.^22^ By analysing their expression profiles in our RNA-seq dataset, we aimed to identify regulatory candidates that might contribute to the splicing alterations observed in heart failure. Only one RBP, *RBM47*, was consistently downregulated in both DCM (*P* = 1.7 × 10^−10^; Log_2_FC = −1.22) and ICM (*P* = 8.9 × 10^−11^; Log_2_FC = −1.18). Additionally, *PCBP3* was downregulated in ICM (*P* = 0.003; Log_2_FC = −1.15), and *HNRNPF* and *MATR3* were downregulated in DCM (*P* = 2.3 × 10^−10^; Log_2_FC = −1.07 and *P* = 1.2 × 10^−4^; Log_2_FC = −1.06, respectively) (see Supplementary material online, *Figure S3F* and *Table S6*). We also examined a manually curated subset of splicing factors implicated in cardiac splicing regulation (see Supplementary material online, *Table S6*) but found no significant differences in their mRNA expression between both patient groups and controls (*Figure 3D*). Among cardiac splicing regulators, RBM20 stands out for its role in cardiomyopathy pathogenesis.^23^ Mutations in *RBM20* are associated with severe familial forms of dilated cardiomyopathy^5^ and RBM20 is known to control the splicing of key cardiac transcripts that are mis-spliced in DCM hearts.^12^ Although *RBM20* mRNA levels tended to be higher in both DCM and ICM samples compared to controls (*Figure 3E*), the increase did not reach statistical significance in DCM (*P* = 5.73 × 10^−7^; Log_2_FC = 0.92). Given that a previous study reported increased *RBM20* expression in DCM hearts,^12^ suggesting that its dysregulated expression may drive disease progression, even in the absence of pathogenic mutations,^12^ we sought to validate this finding in a larger cohort. Using qRT–PCR, we quantified *RBM20* mRNA in LV samples from 54 DCM patients, 45 ICM patients, and 23 controls. No significant differences in *RBM20* expression were observed across the groups (*Figure 3E*), suggesting that changes in the abundance of transcripts encoding splicing regulators, including RBM20, are unlikely to account for the splicing abnormalities observed in heart failure.

To further explore the possibility that splicing alterations in DCM and ICM reflect changes in RBP function, we next searched for enrichment of RBP binding motifs in the vicinity of differentially spliced events identified in failing hearts. We used rMAPS2,^24^ a web-based tool designed to detect enrichment of RBP binding motifs near AS events detected by *rMATS*. For exon skipping events, binding motifs for the cardiac-associated RBP Quaking (*QKI*)^25^ were significantly enriched in the downstream introns of exons that were less included in both DCM and ICM (*P* = 4.39 × 10^−7^ and *P* = 5.74 × 10^−7^, respectively; *Figure 3F, G*). Motif enrichment in both up- and down-regulated exon skipping events was also observed at the upstream exon-intron region of DCM and ICM events for the RBPs *HNRPC*, *HNRPCL1*, *TIAL1*, *HuR*, *RALY*, and *PTBP1*. None of these RBPs were identified as differentially regulated at the RNA level (see Supplementary material online, *Tables S6* and *S7*). *PTBP1,* which shows a more modest enrichment when compared to QKI (see Supplementary material online, *Table S7*), exhibited a trend towards downregulation in the failing heart, which did not meet statistical significance criteria (log_2_FC = −0.81297 and log_2_FC = −0.77782 in DCM and ICM, respectively) (*Figure 3D* and Supplementary material online, *Table S6*). While other cardiac associated RBPs, such as Muscleblind-like splicing regulator 1 (*MBNL1*), showed motif enrichment in upstream intronic region for ICM events, they did not reach significance in both disease aetiologies and within the same genomic region. No consistent significant motif enrichment was observed for other splicing event types across DCM and ICM (see Supplementary material online, *Table S7*). QKI is known to bind intronic regions flanking regulated exons and to modulate the splicing of key cardiac genes such as *ACTN2* and *TTN*,^26^ supporting its potential role in driving exon skipping alterations in the failing heart. Because our RNA-seq analysis did not reveal significant changes in *QKI* mRNA levels (*Figure 3D*), we evaluated QKI protein abundance in a subset of heart biopsies. Consistent with the transcriptomic data, QKI protein expression remained unchanged in DCM and ICM (*Figure 3H* and Supplementary material online, *Figure S3G*). Notably, previous studies have shown that splicing factor activity can be modulated independently of transcript or protein levels, through mechanisms such as post-translational modifications, protein–protein interactions, and changes in subcellular localization.^27^

### 3.3 Conserved splicing dysregulation in heart failure is reproducible across cohorts

Due to the limited number of heart samples analysed by RNA-seq, we aimed to validate whether splicing events identified as dysregulated in DCM and ICM were consistently altered in a larger cohort. First, we found that, despite methodological differences in splicing quantification, approximately 10% of the events identified in our study overlapped with mis-spliced events reported in an independent cohort of 97 DCM heart samples^12^ (see Supplementary material online, *Table S8*). We then selected a representative subset of events, spanning multiple splicing types and a range of ΔPSI values, for validation by qRT–PCR in LV tissue from 54 DCM patients, 45 ICM patients, and 23 non-failing controls (*Figure 1C*). To prioritize splicing events for experimental validation, we selected those that were differentially regulated in both DCM and ICM (see Supplementary material online, *Figure S4* and *Table S3*) and whose genomic coordinates overlapped with those reported in the previous DCM study.^12^ We focused on genes with well-established roles in cardiac physiology and initially analysed binary exon skipping events that presented similar ΔPSI changes in both DCM and ICM hearts (*CAMK2D* and *MYL6*). In addition, we identified a highly downregulated exon-skipping event in *PDLIM3* that showed consistent and robust regulation in our HF samples (see Supplementary material online, *Figure S4A* and *B*). Using *MAJIQ*, we selected three complex splicing events in *EYA4, SORBS1*, and *ESRRG.* The genomic coordinates of these events matched those identified in the previously published DCM dataset that reported differential exon usage but did not resolve the full complexity of the underlying splicing patterns,^12^ making these events strong candidates for experimental validation (see Supplementary material online, *Figure S4C*—*H*).

**Figure 4:**
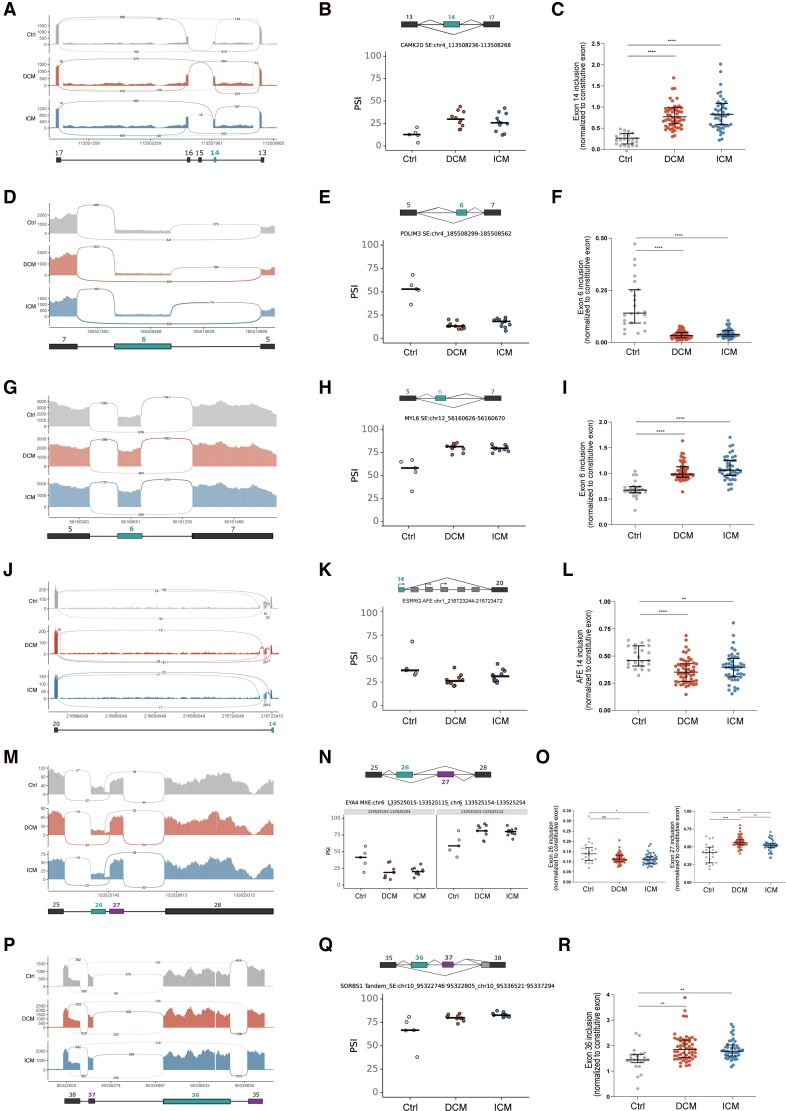
Validation of selected dysregulated splicing events. For each mRNA, the dysregulated alternative exon is highlighted. In *ESRRG*, an arrow denotes the transcription start site (AFE, alternative first exon). Left panels: Sashimi plots. Middle panels: PSI values from RNA-seq data. Right panels: qRT-PCR quantification of target exon inclusion normalized to the level of a constitutive exon from the same gene (*Figure 1*C) across the entire cohort (*n* = 54 DCM, *n* = 45 ICM and *n* = 23 controls), with statistical significance indicated as follows: *ns* (*P* > 0.05), *P < 0.05, **P < 0.01 and ****P < 0.0001. Statistical analysis was performed using Brown-Forsythe and Welch ANOVA tests to account for unequal variances, followed by Games-Howell's multiple comparisons test.

The *CAMK2D* gene encodes Ca^2+^/calmodulin-dependent protein kinase II delta (CAMK2δ), a key regulator of cardiac intracellular calcium handling and signalling.^28^ AS of exons 14, 15, and 16 in *CAMK2D* pre-mRNA generates three main isoforms in the human heart: CaMKIIδB (exons 13, 14, 17), CaMKIIδC (exons 13, 17), and CaMKIIδ9 (exons 13, 16, 17).^29^ Our analysis revealed a consistent increase in the inclusion of exon 14 (chr4:113508236–113508268) in both DCM and ICM hearts compared to controls (*Figure 4A, B*). This was detected in RNA-seq data using *rMATS*, showing a ΔPSI of 0.416 in DCM and 0.401 in ICM, as well as *vast-tools*, with a ΔPSI of 0.39 in DCM and 0.33 in ICM (see Supplementary material online, *Table S3*). To validate this splicing event, we designed primers to distinguish transcripts including or excluding exon 14 of *CAMK2D* by RT-PCR (see Supplementary material online, *Figure S5A*). Quantification by qRT–PCR in the extended cohort confirmed significantly higher inclusion of exon 14 in failing hearts compared to controls (*Figure 4C*), consistent with the RNA-seq findings. Exon 14 of *CAMK2D* encodes an 11-amino acid sequence that contains the nuclear localization signal (NLS) required for directing CaMKIIδ to the nucleus.^30^ Consequently, the CaMKIIδB isoform, which includes exon 14, can translocate to the nucleus and modulate calcium-dependent gene expression programmes.^31^ In parallel to a consistent increase in exon 14 inclusion observed in HF samples, we also detected a significant reduction in the inclusion of exon 16 (chr4:113502936–113502977) in both DCM and ICM hearts compared to controls. This was supported by *rMATS* analysis, which showed a ΔPSI of −0.251 in DCM and −0.218 in ICM, and by *vast-tools*, which reported ΔPSI values of −0.17 in DCM and −0.16 in ICM (see Supplementary material online, *Figure S5B* and *Table S3*). Since exon 16 is a defining feature of the CaMKIIδ9 isoform, these findings suggest decreased expression of CaMKIIδ9 in failing hearts.

The *PDLIM3* gene encodes Alpha-Actinin-2-Associated LIM Protein (ALP), a member of the PDZ and LIM domain protein family that interacts with α-actinin and plays a crucial role in maintaining cardiac muscle structure and function.^32^ Exon 6 of *PDLIM3* undergoes AS, although its specific functional significance remains unclear.^33^ We observed significantly reduced inclusion of exon 6 (chr4: 185508562–185508299) in both DCM and ICM hearts compared to controls (*Figure 4D, E*). This splicing alteration was detected in RNA-seq data using *rMATS* with a ΔPSI of −0.394 in DCM and −0.964 in ICM (see Supplementary material online, *Table S3*) and subsequently validated by RT–PCR (see Supplementary material online, *Figure S5C*) and qRT–PCR (*Figure 4F*). *PDLIM3* exon 4 (chr4: 185514890–185514703) can also be alternatively spliced.^34^ However, we found no significant changes in the levels of exon 4 inclusion between control and HF hearts (see Supplementary material online, *Figure S5D*).

The *MYL6* gene encodes a ubiquitously expressed myosin light chain subunit that undergoes cell-type-specific AS.^35^ We observed a significant increase in the inclusion of *MYL6* exon 6 (chr12:56160626–56160670) in both DCM and ICM hearts compared to controls (*Figure 4G, H*). This previously uncharacterized change was identified in RNA-seq data using *rMATS* (ΔPSI: 0.25 in DCM and 0.24 in ICM) and *MAJIQ* (ΔPSI: 0.13 in DCM and 0.16 in ICM) (see Supplementary material online, *Table S3*). This finding was further validated by RT–PCR (see Supplementary material online, *Figure S5E*) and qRT–PCR (*Figure 4I*).

The *ESRRG* gene encodes Estrogen-Related Receptor Gamma (ERRγ), a key regulator of cardiomyocyte maturation and a transcriptional activator of genes essential for adult cardiac metabolism and structure.^36^ ERRγ signalling is crucial for driving the expression of genes involved in mitochondrial function and cardiac-specific contractile processes.^37^ Previous reports demonstrated that *ESRRG* AS results in mRNAs with different 5'-ends.^38^ Our analysis using *MAJIQ* identified a region between exons 10 and 20 containing multiple alternative first exons (see Supplementary material online, *Figure S5F*). A particular exon was differentially regulated in control and HF samples, reported as part of two distinct alternative first exon events (see Supplementary material online, *Figure S5F*) and corresponding to an *ESRRG* isoform that starts at exon 14 (chr1:216723429–216723244). RNA-seq data revealed a significant reduction in the abundance of transcripts containing exon 14 joined to exon 20 in both DCM and ICM hearts compared to controls (*Figure 4J, K*). To validate this finding, we designed primers targeting exon 14 and exon 20 for qRT–PCR (*Figure 4L*). Quantification confirmed the reduced expression of this *ESRRG* isoform in failing hearts, consistent with the RNA-seq results.

The *EYA4* gene encodes EYA Transcriptional Coactivator and Phosphatase 4, a transcriptional coactivator essential for normal heart function. Mutations in this gene have been linked to dilated cardiomyopathy and heart failure.^39^ A previous study reported that *EYA4* exons 26 and 27 are both expressed in adult heart, whereas only exon 27 is detected in skeletal muscle, suggesting cardiac tissue specificity of exon 26.^40^ Our analysis using *MAJIQ* identified exons 26 and 27 as mutually exclusive, with increased inclusion of exon 27 (chr1:58668430–58668566) and a corresponding skipping of exon 26 (chr6:133525015–133525115) in DCM (ΔPSI: 0.22) and ICM (ΔPSI: 0.21) (*Figure 4M, N*). These findings were independently validated by qRT–PCR (*Figure 4O*).

The protein encoded by the *SORBS1* gene (Sorbin And SH3 Domain Containing 1) is a cytoskeleton adaptor protein required for angiogenesis and lymphangiogenesis.^41^ Our analysis using *MAJIQ* detected a tandem cassette exon skipping event in which exons 36 and 37, located in the terminal region of *SORBS1* transcripts, are either jointly skipped or included with ΔPSI values of 0.16 in ICM and 0.13 in DCM (*Figure 4P, Q*). Although *SORBS1* splicing isoforms have been previously described^42^ this specific splicing event has not been characterized. For validation, we performed RT–PCR (see Supplementary material online, *Figure S5G*) and qRT–PCR (*Figure 4R*) to assess the differential splicing of each exon involved in this event individually. Quantification in the larger cohort confirmed a significant increase in the inclusion of exons 36 (chr10:95336521–95337294) and 37 (chr10:95322746–95322805) in both DCM and ICM hearts (*Figure 4R*).

Next, we investigated potential RBP interaction sites in the regions surrounding the AS events validated in *CAMK2D, PDLIM3, MYL6*, *EYA4*, and *SORBS1*. Due to the high complexity of the splicing pattern, this analysis was not performed for the *ESRRG* event. We used *catRAPID omics v2.0*,^43^ a computational tool that predicts protein-RNA interactions based on physicochemical properties and secondary structure. We found a high predicted binding score (rank >0.8) for QKI near the alternative exons of *EYA4*, *PDLIM3*, and *SORBS1*. In addition, high binding propensities were predicted for HNRNPU and members of the RNA Binding Motif family, including RBM10 and RBM15, in the vicinity of alternative exons in *CAMK2D*, *MYL6*, *PDLIM3*, and *SORBS1*. A complete list of the RBPs predicted to interact with these regions is provided in Supplementary material online, *Table S9*.

To explore the potential clinical significance of these splicing alterations, we next analysed correlations between exon inclusion levels (assessed by qRT–PCR) and key cardiac parameters associated with disease severity, including cardiac index, ejection fraction, and left ventricular end-diastolic diameter, using available clinical metadata from heart failure patients (see Supplementary material online, *Table S1B*). No significant correlations were observed (see Supplementary material online, *Figure S6*), suggesting that the splicing changes identified may reflect common molecular changes of cardiac performance at advanced disease stages.

### 3.4 Multi-chamber analysis uncovers differential spatial distribution of splicing events in heart failure

To investigate whether the observed splicing changes were confined to the LV, which is the primary heart chamber affected by cardiac remodelling during HF progression^44^ we extended the qRT–PCR analysis to matched samples from the RV and IVS of 21 controls, 25 DCM, and 15 ICM patients (*Figure 1D*).

**Figure 5:**
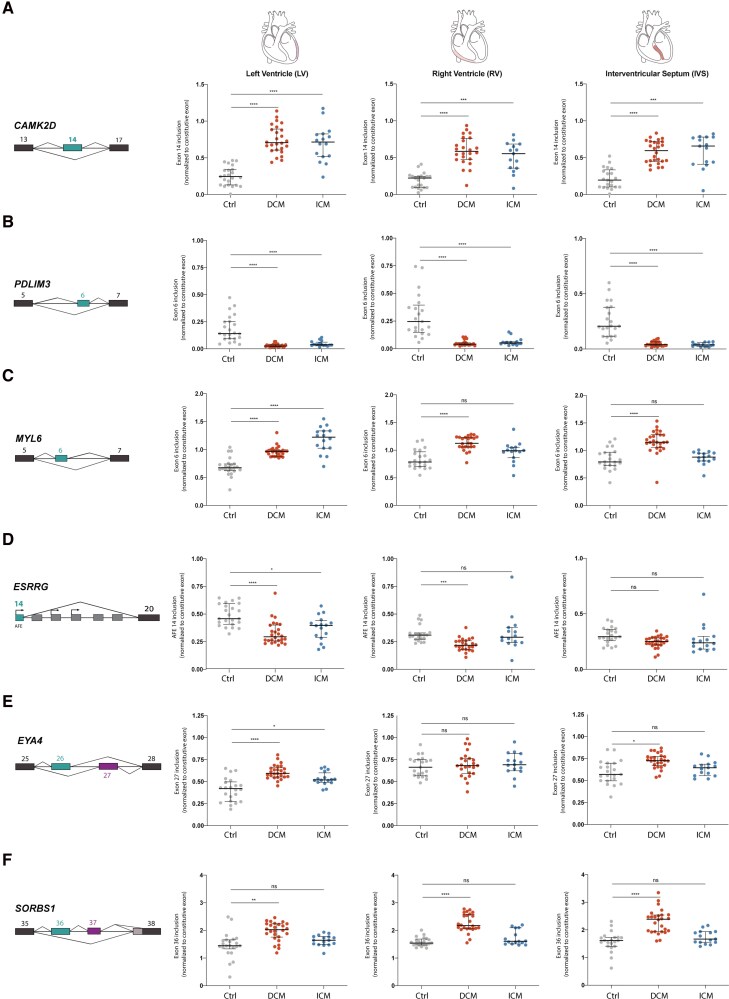
Dysregulated splicing across cardiac regions. For each mRNA, the dysregulated alternative exon is highlighted. Results of qRT-PCR analysis from the indicated regions of the heart (21 controls, 25 DCM, and 15 ICM patients). Statistical significance is indicated as follows: *ns* (*P* > 0.05), *P < 0.05, **P < 0.01, and ****P < 0.0001. Statistical analysis was performed using Brown-Forsythe and Welch ANOVA tests to account for unequal variances, followed by Games-Howell's multiple comparisons test.

For *CAMK2D* exon 14 and *PDLIM3* exon 6, similar alterations were observed across the LV, RV, and IVS in both DCM and ICM samples, indicating widespread splicing dysregulation of these mRNAs in failing hearts (*Figure 5A, B*).

For *MYL6* exon 6, significantly higher inclusion levels were detected across the LV, RV, and IVS in DCM samples (*Figure 5C*). However, in ICM hearts, exon 6 inclusion was significantly higher only in the LV, whereas inclusion levels in the RV and IVS remained similar to those of controls (*Figure 5C*).

In DCM hearts, inclusion of *ESRRG* exon 14 was significantly reduced in the LV and RV compared to controls, while inclusion levels in the IVS remained comparable to those in controls (*Figure 5D*). In ICM hearts, a significant reduction in exon 14 inclusion was observed only in the LV, with no notable changes in the RV or IVS (*Figure 5D*).

For *EYA4* exon 27, DCM hearts exhibited significantly higher inclusion levels in the LV and IVS, while no significant changes were observed in the RV (*Figure 5E*). In ICM hearts, increased exon 27 inclusion was detected exclusively in the LV, with no significant differences in the IVS or RV (*Figure 5E*).

In DCM hearts, inclusion of *SORBS1* exon 36 was significantly increased across the LV, RV, and IVS compared to controls (*Figure 5F*). In contrast, no significant differences were detected in ICM hearts (*Figure 5F*). However, in the larger cohort, a significant increase in exon 36 inclusion was detected in the LV of ICM samples (*Figure 4R*).

Taken together, these results identify splicing events that exhibit widespread dysregulation across cardiac compartments in failing hearts, regardless of aetiology. Other splicing alterations show distinct patterns of distribution. Splicing dysregulation in ICM appears more frequently confined to the LV, whereas DCM is characterized by more extensive and pervasive splicing changes across the heart.

## 4. Discussion

This study provides a comprehensive analysis of AS in human heart failure and reveals both shared and spatially distinct patterns of dysregulation in end-stage dilated (DCM) and ischaemic cardiomyopathy (ICM). By analysing RNA-seq data from LV tissue using three complementary computational tools, *rMATS*, *vast-tools*, and *MAJIQ*, we identified numerous dysregulated splicing events present in both DCM and ICM hearts. Considering the intrinsic differences in event detection across splicing tools,^14–16^ our approach uncovered a previously uncharacterized set of complex AS events dysregulated in HF. In addition to exon skipping, alternative first and last exons were among the most frequently dysregulated events and have the potential to substantially increase isoform diversity and impact protein function by altering *N*- and *C*- terminal regions.^45^

The convergence of splicing alterations across disease aetiologies mirrors findings from prior transcriptomic studies showing similar gene expression patterns in DCM and ICM.^7,8^ Together, these results reinforce the concept that HFrEF elicits stereotyped molecular responses, encompassing both transcriptional and post-transcriptional changes, regardless of initial cause.

Our analysis also revealed that transcriptional dysregulation is more pervasive than splicing dysregulation in failing hearts, as the number of differentially expressed genes exceeded the number of differentially spliced events. This suggests that while transcriptional responses may broadly reflect pathological remodelling, splicing changes may act as more selective modulators of protein function, potentially fine-tuning the myocardial response to chronic stress.

Notably, while this manuscript was under revision, a study by Haas et al. was published describing long-read nanopore sequencing of LV tissue from non-failing controls, and patients with DCM and ICM.^46^ Their study revealed widespread isoform switching across sarcomere genes and showed that the overall isoform landscapes in DCM and ICM were largely indistinguishable. These findings are in strong agreement with our results and further support the notion that end-stage HF is characterized by a convergent splicing landscape, irrespective of disease aetiology.

Although RBPs are central to splicing control, our expression analysis revealed minimal changes in RBP transcript levels in DCM and ICM compared to controls. This includes QKI, whose RNA and protein levels were unchanged. These observations argue against a model in which altered RBP abundance is the primary driver of splicing defects in heart failure, and instead point to alternative mechanisms, such as changes in RBP activity, protein–protein interactions, subcellular localization, or post-translational modifications that modulate splicing outcomes without altering overall expression levels.^47–49^ In particular, phosphorylation has been shown to regulate the splicing activity of several RBPs, including RBM20, a key modulator of cardiac splicing.^50–52^ Elucidating upstream signalling pathways that modulate RBP function, as well as understanding the roles of RNA secondary structure, transcription kinetics, and chromatin modifications in splice site selection^9,27^ will be crucial to fully understand the mechanisms of splicing dysregulation in heart failure.

Motif enrichment analysis of differentially spliced regions implicated QKI as a major candidate splicing regulator in both DCM and ICM failing hearts. QKI has been shown to modulate the splicing of *CAMK2D* and to bind intronic regions of other cardiac-relevant transcripts such as *PDLIM5*.^25,26^ RBM20 is another known regulator of *CAMK2D*, *PDLIM3*, and *SORBS1* isoforms.^50,51^ However, RBM20 was not among the top hits in our *catRAPID* predictions, and its motifs were not included in *rMAPS2* analyses, underscoring the limitations of current computational tools and the need for expanded motif datasets for cardiac-enriched RBPs.

A major strength of our study is the validation of splicing events in an expanded cohort of 122 heart samples. We showed increased inclusion of *CAMK2D* exon 14, which encodes an NLS, in both DCM and ICM samples compared to controls. This observation aligns with a previous study reporting increased inclusion of *CAMK2D* exon 14 in DCM hearts.^53^ Additionally, a shift from *CAMK2D* isoforms lacking exon 14 to those including it has been associated with activation of hypertrophic signalling in animal models.^54,55^ We also discovered novel dysregulation of *PDLIM3* exon 6 inclusion in both DCM and ICM. This exon encodes a ZASP-like motif that may interact with α-actinin and influence sarcomeric function.^32^ No changes were observed in exon 4 inclusion, which is included in the predominant isoform detected in the heart.^34^ Additional splicing changes were observed in *MYL6*, where increased inclusion of exon 6, encoding part of an EF-hand protein domain, may affect calcium-binding function.^35^ In *ESRRG,* which encodes a transcriptional regulator essential for cardiac maturation and adult heart function,^36^ we identified downregulation of isoforms initiating at exon 14 in both DCM and ICM. This represents a previously uncharacterized switch that may impair cardiac transcriptional programs. *EYA4*, a gene associated with familial DCM,^39^ showed increased inclusion of exon 27, revealing a potentially functionally distinct isoform in failing hearts. Lastly, *SORBS1*, known to regulate cytoskeletal and angiogenic processes, exhibited increased inclusion of exons 36 and 37 in both DCM and ICM, linking this adaptor protein to heart failure for the first time at the splicing level.^41^ Notably, *Sorbs2* knockout in mice results in DCM.^56^

An important novel aspect of our study is the spatial analysis of splicing dysregulation across the heart. Because the LV is the primary cardiac chamber affected in both DCM and ICM,^44^ the role of the RV in heart failure has received comparatively less attention. However, the growing recognition of RV dysfunction as a critical determinant of HF progression and outcomes underscores the importance of investigating molecular alterations in this chamber.^57^

Although we observed some splicing events (e.g. in *CAMK2D* and *PDLIM3*) dysregulated across all chambers in both DCM and ICM, for other genes, namely *MYL6*, *ESRRG*, *SORBS1*, and *EYA4*, we identified a fundamental difference in the pattern of splicing dysregulation between DCM and ICM. In DCM, splicing alterations consistently affect all cardiac regions, suggesting a more diffuse cardiomyopathic process. In contrast, the chamber-specific splicing dysregulation observed in ICM may reflect the localized nature of ischaemic injury.

Translational perspective This study identifies aberrant AS as a shared molecular hallmark of both dilated and ischaemic cardiomyopathy in end-stage heart failure. By mapping splicing alterations across multiple cardiac regions, we uncover distinct spatial patterns of dysregulation, highlighting chamber-specific differences that may reflect underlying disease mechanisms. These findings lay the groundwork for future efforts to link regional splicing profiles with clinical phenotypes, which could ultimately inform the development of more precise diagnostic tools and targeted therapeutic strategies in heart failure.

## 5. Conclusions

This study provides new insights into the role of AS in heart failure, identifying numerous dysregulated splicing events shared between DCM and ICM. The convergence of splicing patterns across aetiologies suggests that splicing dysregulation is a common molecular response to pathological cardiac remodelling in HFrEF. At the same time, the identification of spatially distinct splicing alterations highlights the potential contribution of isoform switching to disease heterogeneity and progression. Future studies integrating the functional characterization of specific isoforms and mechanistic dissection of the signalling pathways that modulate splicing regulators will be essential to elucidate the impact of AS on HF pathogenesis and to explore its potential as a target for diagnosis or therapy.

## 6. Limitations of the study

A limitation of this study is the relatively small number of samples analysed, particularly in the RNA-seq cohort, and the limited number of splicing events validated by qRT–PCR. This may restrict the generalizability of our findings and reduce the statistical power to detect more subtle splicing alterations. For example, while increased inclusion of *SORBS1* exon 36 was significant in the cohort of 45 ICM samples, this difference was not detected in a smaller subset of 15 samples. Additionally, our splicing analysis was based on an event-centric approach that quantifies exon inclusion or exclusion but does not capture the full-length isoform architecture. The use of short-read RNA-seq with 75 bp read lengths may also have constrained the accuracy of splice junction mapping. Finally, the predominance of male patients in our cohort limits the ability to assess sex-specific differences in splicing regulation. Future studies using long-read sequencing technologies and larger, more diverse cohorts will be essential to enable full-length transcript reconstruction and to better capture inter-individual and sex-related variability in splicing patterns.

## Supplementary Material

cvag068_Supplementary_Data

## Data Availability

RNA-seq data are deposited at the GEO repository under accession number GSE165556.
