# Supplementary material for: Shared splicing dysregulation in heart failure associated with dilated and ischaemic cardiomyopathy and spatial specificity across cardiac regions
Source: Cardiovasc Res. 2026 Mar 24;122(7):906–18. doi: 10.1093/cvr/cvag068 (PMC13196872; doi:10.1093/cvr/cvag068)
Supplement: cvag068_Supplementary_Data [file cvag068_supplementary_data.zip › Supplementary Material and Figures_Table S1 and S2.docx]

**Supplementary Material**

**Supplementary Methods**

**RNA isolation**

Total RNA isolation and purification from human hearts followed the methodology presented in Firat H., Zhang L. *et al*., 2023^1^, using the mirVana isolation kit (Life technologies, Merelbeke, Belgium) and DNase I (Qiagen, Venlo, The Netherlands) digestion according to the manufacturer’s instructions. RNA concentration was measured with Nanodrop (Nanodrop products, Wilmington, USA) and RNA integrity was analysed using a 2100 Bioanalyzer (Agilent technologies, Santa Clara, USA).

**RNA sequencing (RNA-Seq)**

The Illumina TruSeqTM stranded mRNA and total RNA kit was used for library preparation. Ribosomal RNAs were removed using the RiboZero ribosomal RNA removal kit (Illumina Inc. San Diego, USA). All steps were performed according to the manufacturer’s instructions. The ribosomal RNA-depleted RNA samples were fragmented by heat digestion with divalent cations (8 min at 94 °C) and reverse transcribed into cDNA using Superscript II reverse transcriptase (Thermo Fisher Scientific, Luxembourg). Following second strand synthesis, the resulting blunt-ended cDNA was purified using AMPure XP magnetic beads.  Illumina's adapter ligation was performed after 3'end adenylation and the single indexed libraries were amplified by PCR (15 cycles). 1 μl of each library was run on the Agilent Technologies 2100 Bioanalyzer and all libraries were sequenced with the Illumina NextSeq500. Most samples were sequenced with 75 bp paired-end reads, while one run used 100 bp paired-end reads for samples F1505924, F1505939, and F1505940.

**Data preprocessing, transcript quantification, and genome alignment**

Raw data was preprocessed with TrimGalore v0.6.10 ^2^ to remove possible sequencing adapters and filter sequences by Phred quality scores (“*-q 20 --length 60 --stringency 5 --trim-n --max_n 2, --clip_R1 1 --clip_R2 1 --three_prime_clip_R2 1 --three_prime_clip_R2 1 --paired*”). Due to the high levels of rRNA in some samples, we ran the kmer-based BBDuk utility from BBTools v39.03 ^3^ suite to filter out those sequences based on a list of rRNA sequences from the GRCh38 genome build using a k-mer size of 31. Transcript-level quantifications were obtained with Salmon v1.10.2^4^ (“*--validateMappings --minScoreFraction 0.65 --numBootstraps 100 --seqBias --gcBias --fldMean 200 --fldSD 50 --libType A*”) against the GENCODE human v44 transcriptome^5^. Genome alignments were performed with STAR 2.7.10b^6^ against the GRCh38 primary genome assembly using the GENCODE v44 reference annotations (“*--twopassMode basic --chimOutType WithinBAM SoftClip --outFilterScoreMinOverLread 0.4 --outFilterMatchNminOverLread 0.4 --outFilterMatchNmin 60 --quantMode GeneCounts --outSAMtype BAM SortedByCoordinate*”). Uniquely mapped reads were obtained (mapping quality value of 255) and additional quality controls with MultiQC v1.15^7^ and RSeQC v5.01^8^ were conducted. In particular, read alignments in rRNA genome locations were removed (RSeQC split_bam.py utility), with the resulting BAM files selected for downstream analysis.

**Gene expression analysis**

We obtained gene-level counts from Salmon quantifications using tximport v1.32.0^9^ (“type=’salmon’, txIn=T, txOut=F, countsFromAbundance=’no’”), and low counts were pre-filtered by only keeping genes with more than 10 reads in at least 10 samples. Differential gene expression analysis between patients and controls was performed using DESeq2 v1.44.0^10^. A multi-factor design was incorporated (*~sex + group*) to account for sample gender while estimating the group effect (Control, DCM, ICM). Wald test was employed for differential testing and effect size estimates were shrunken with the apeglm method^11^. Genes with log2FoldChange > 1 and FDR-adjusted P-value < 0.05 were considered differentially expressed. To inspect gene expression of RNA-binding proteins (RBPs) involved in RNA Splicing, we retrieved RBPs identified in ENCODE^12^ based on their assigned function flags (Splicing regulation=1 or Spliceosome=1, n=111). The normalized expression values (DESeq2’s median of ratios) for these genes were used to draw an expression heatmap of splicing-associated RBPs across samples. The heatmap was created using the ComplexHeatmap v2.20.0 package^13^.

**Splicing analysis**

***rMATS*** was run with two different modes, one to quantify inclusion levels across all samples (with all BAM files provided in *--b1* and *--statoff* set) to be further used for dimensionality reduction analysis by principal component analysis (PCA), and the other to perform differential splicing analysis between pairs of groups (*--b1* and *--b2* set, *--cstat 0.05*). The following parameters were common to both modes: *-t paired --anchorLength 3 --task both –readLength 75 --variable-read-length --allow-clipping --libType fr-firststrand*. The GENCODE v44 primary assembly GTF was used as the gene and transcript annotations. For PCA, we filtered splicing quantifications to retain only events where the isoform with the most reads (inclusion or exclusion) had a median read coverage of at least 15 across all samples. For differential splicing analysis, we considered events significant if they met all of the following criteria: an absolute dPSI ≥ 0.1 between the two conditions, an FDR ≤ 0.05, and an average junction read count ≥ 5 in both conditions for the isoform with more reads (either inclusion or exclusion).

***vast-tools*** uses its own annotation from VastDB database. We used the vastdb.hs2.23.06.20 version for our analysis. Preprocessed reads (BBDuk filtered) were input to *vast-tools align* (*--IR_version 2 --keep*), and PSI quantifications were retrieved by *vast-tools tidy* (*--noVLOW --min_SD -min_Fr 0.75*) from the inclusion levels’ file generated with *vast-tools combine*. The tidy table was directly used for dimensionality reduction analysis. Differential splicing analysis was conducted with *vast-tools diff* using *--prob 0.9 --minDiff 0.1 --minSamples 3 --minReads 7*. An event was considered significant if both the absolute estimated dPSI and the MV at 0.9 probability were larger than 0.1, and the event passed the coverage filters employed in *vast-tools tidy*.

***MAJIQ****’s* *build* module was run with the GENCODE v44 primary assembly GFF3 as the genome annotations, with the following parameters: *--min-experiments 4 --minreads 5 --minpos 3 --min-denovo 7 --irnbins 0.5 --min-intronic-cov 0.2 --ext3prime 2000 --ext5prime 2000*. The *psi* module was used to quantify per-sample inclusion levels of splice junctions for each Local Splicing Varation (LSV), retaining LSVs with *--minreads 10* and *--minpos 3*. These results were further processed with *voila modulize* (*--show-read-counts --show-per-sample-psi --show-all --untrimmed-exons --decomplexify-psi-threshold 0 --decomplexify-reads-threshold 1*) to generate splicing quantifications for classical alternative splicing events. These tables were used for dimensionality reduction analysis, with the AS modules containing de novo junctions discarded. In addition, we retained only the AS modules for which the median coverage across all samples for the isoform with the most reads (inclusion or exclusion-supporting junctions) was larger than 15. For each event, we selected the junction supporting the inclusion isoform with the largest variance as the single PSI value for each event (e.g., for exon skipping, the junction with *spliced_with = “A”* that had the largest variance). The *heterogen* module was employed to detect differentially spliced events in pairwise comparisons among the controls, ICM, and DCM groups by requiring at least 4 samples per group (*--min-experiments 4*) to pass per-experiment filters (*--minreads 10 --minpos 3*). Consequently, we ran *voila modulize* (*--show-read-counts --show-per-sample-psi --show-all --untrimmed-exons –decomplexify-psi-threshold 0.05 --decomplexify-deltapsi-threshold 0.01 --decomplexify-reads-threshold 5*) on the *heterogen* output to process LSVs into AS modules that can be classified into subtypes representing classical alternative splicing events. We further processed the results to produce tables comparable to other tools. In particular, for classical AS events (exon skipping, intron retention alternative 3’ and 5’ splice sites, and MXE), we kept only modules with one splicing event (binary splicing decisions). To better represent biological complexity, we took advantage of *MAJIQ’s* capabilities and included *de novo* events containing splice junctions absent from the genome annotations. In addition, we processed three additional event types that neither *rMATS* nor *vast-tools* detect: alternative first exons, alternative last exons, and tandem exon skipping (2 or more alternative exons skipped/included together). For these, we allowed them to be part of complex modules with multiple event types. Because *modulizer* displays a dPSI estimate for each module’s junction, we processed each event type to assign a single dPSI for each event. For exon skipping and tandem exon skipping, we selected the junction supporting the inclusion (*spliced_with = “A”* for exon skipping and *spliced_with = “A1|A_Last”* for tandem exon skipping) with the largest absolute dPSI change.  For intron retention, we selected the junction supporting the retention (*junction_name = “C1_C2_intron|C2_C1_intron”*). For MXE, we assigned the first alternative exon as the inclusion form (just like rMATS does) by extracting the corresponding junction with the largest absolute dPSI change (*junction_name = “C1_A1|C2_A1”*). For alternative 3’ and 5’ splice sites, the dPSI referred to the junction supporting the longest isoform (*junction_name = “Proximal”*). Lastly, for alternative first and last exon events, we retrieved the junction from the exon with the largest dPSI change. Regardless of the event type, an event was considered significant if it met all the following criteria: the absolute dPSI ≥ 0.1, the Mann-Whitney U p-value ≤ 0.05, and the group median number of reads in both conditions for the selected junction was larger than 5.

**Dimensionality Reduction Analysis**

We performed PCA on splicing quantifications processed for each tool. Before PCA, we applied a filtering criterion where events with missing quantifications in more than one sample were discarded. For events missing quantifications in exactly one sample, we performed imputation using the median PSI value of the group to which the sample belonged. Finally, the 1500 events with the highest variance across samples were used for PCA. The analysis was conducted using the scikit-learn v1.3.1 implementation^14^.

**Enrichment analysis**

Enrichment analysis in differentially spliced genes and differentially expressed genes was performed using the *gost* function from the gprofiler2^15^ package in R. The analysis included Biological Process (BP) and for each enriched term we calculated the gene ratio, defined as the proportion of enriched genes associated with a given GO term, relative to the total number of genes associated with that pathway. The function was run separately for each tool and comparison. Background gene sets were defined using normalized counts from DESeq2, including genes with >10 reads in at least 7 samples (DCM vs. Ctrl, ICM vs. Ctrl) or 8 samples (DCM vs. ICM). Multiple testing correction was applied using the default g:SCS method; we used *domain_scope = "custom_annotated"*; and only terms with an adjusted p-value < 0.05 were considered significant.

**Volcano Plots**

Visualization of alternative splicing events was performed per tool and per event type. Volcano plots were generated using *ggplot2*, with ΔPSI plotted on the x-axis and –log10(p-value) on the y-axis. Vertical dashed lines at ΔPSI = –0.1 and +0.1 indicated the biological effect-size thresholds. An event was classified as *significant* if it met both criteria: Mann-Whitney U p-value ≤ 0.05 and |ΔPSI| ≥ 0.1 for *MAJIQ* and FDR ≤ 0.05 and ΔPSI| ≥ 0.1 for *rMATS*; all remaining events were labeled as *not significant*. The specific alternative splicing events validated in the extended cohort are highlighted in blue.

**Overlap of Splicing Events Across Tools**

To identify common splicing events detected by the three tools, we used the curated list of differentially spliced events (**Table S3**) as a starting point and focused on four classical splicing categories: RI, ES, A3SS, and A5SS. MAJIQ events with "True" in the *denovo_event* column were excluded to ensure consistency across tools. Overlaps were assessed using the *target_coordinates* field, but differences in event annotation formats required category-specific handling.

For RI events, we derived *rMATS* intron positions from the *event_ID*, which contains the up and downstream exon coordinates; the intron start was defined as the position immediately after the upstream exon (+1), and the end as the position immediately before the downstream exon (−1). For ES events, coordinates formatting was consistent across tools, allowing direct comparisons. For A3SS and A5SS events, *rMATS* and *MAJIQ* report both long and short splice site forms as a pair of coordinates separated by a semicolon in *target_coordinates*, whereas vast-tools reports only one of these forms. Therefore, we split the coordinate pairs from *rMATS* and *MAJIQ* and considered an event overlapping with *vast-tools* if either coordinate matched.

**Sashimi Plots**

Sashimi plots for validated splicing events were generated using *ggsashimi* v1.1.5^16^, with *--min-coverage 14*, *--shrink* and *--aggr median*. Annotations were based on the GENCODE v44 primary assembly GTF. For certain comparisons, the y-axis was fixed across tracks (*--fix-y-scale*) to aid visual interpretation.

**Motif enrichment analysis**

Splicing motif analysis was performed using *rMAPS2* (<https://rmaps.cecsresearch.org>), a web-based platform that evaluates the enrichment of RBP motifs surrounding alternative splicing events. *rMAPS2* integrates CLIP-seq datasets of over 100 well-characterized RBPs. The direct output files from *rMATS* are used as input, including all the events identified in the pairwise comparisons DCM vs Controls and ICM vs Controls. For each comparison and event type, *rMAPS2* tested whether specific RBP binding motifs were significantly enriched near differentially spliced exons, using non-regulated events as the background. The tool provides statistical assessments of RBP motif enrichment across multiple regions, including the target exons, flanking introns (upstream and downstream), and exon–intron boundaries. The output is provided separately for each event type, distinguishing between upregulated and downregulated splicing events in HF. The results are presented in **Table S7**.

To identify RNA-binding proteins (RBPs) related to our candidate splicing sites, we used the *catRAPID omics* web server (<http://service.tartaglialab.com>). We predicted interactions between the RNA-binding proteome and pre-mRNA sequences extracted from the genomic coordinates of the splicing site candidates. Results were prioritized based on their ranking scores, with values above 0.8 defined as high-confidence predictions.

**Western Blot**

Protein concentrations were determined using the Bicinchoninic Acid (BCA) assay and 20 µg of protein lysate was loaded per well in 4-20% Mini-PROTEAN precast gels (Bio-Rad, cat # 4561094). Proteins were separated at 60 V for 40 mins then 100 V for 80 mins before gels were blotted onto polyvinylidene fluoride membranes (Immobilon-P, Merck Millipore, cat # IPVH00010) for 60 mins at 100 V. Membranes were then washed twice with deionized water before being incubated with a total protein reagent (No-StainTM Protein labelling reagent, ThermoFisher Scientific, cat # A44717) for 10 mins. Following this, membranes were washed with Tris-buffered Saline with Tween 20 (TBS-T), before being blocked for 60 mins in blocking buffer (5% non-freeze-dried Milk in TBS-T). Membranes were then incubated with a Rabbit monoclonal antibody to QKI overnight at 4 degrees (1/1000 dilution, anti-QKI [EPR7306], Abcam, ab126742). The following day, membranes were washed (3 x 5 mins) in TBS-T before being incubated with a Goat anti-Rabbit secondary antibody (1/20,000 dilution, Jackson Laboratories) for 60 mins at room temperature. Subsequently, membranes were washed and protein of interest and total protein for normalization were revealed using a chemiluminescent substrate (PierceTM ECL Western blotting substrate, ThermoFisher Scientific, cat # 32106). Western blot images were taken and analysed using an Invitrogen iBright Imaging station and analysis software. QKI expression levels were normalized against total protein amounts.

**Quantitative RT-PCR (qRT-PCR)**

We performed cDNA synthesis from 600ng of total RNA using oligodT primers and the Transcriptor High Fidelity cDNA Synthesis Kit (Roche®), according to manufacturer’s instructions. For RT-PCR, regions of interest were amplified using the primers described in **Table S2A** and NZYLong DNA Polymerase (NZYtech®); the amplicons were run on ethidium bromide agarose gels. Quantitative RT-PCR was performed with using the primers described in **Table S2B** and iTaq Universal SYBR Green Supermix (Bio-Rad®) in the ViiA™7 RT-PCR Systems (Applied BioSystems). The relative level of differentially spliced exon in transcripts from a particular gene was quantified using the 2–ΔCt method. The level of the target exon was normalized to the level of a constitutive exon from the same gene (i.e., an exon that is consistently included in all splicing isoforms transcribed from the gene). For statistical analysis we used Brown-Forsythe and Welch ANOVA tests to account for unequal variances, followed by Games-Howell’s multiple comparisons test.

**References**

1. Firat H, Zhang L, Baksi S, Leszek P, Schordan E, Ounzain S, Kottwitz J, Patriki D, Heidecker B, Lüscher TF, Pedrazzini T, Devaux Y. FIMICS: A panel of long noncoding RNAs for cardiovascular conditions. *Heliyon* 2023;**9**:e13087.

2. Martin M. Cutadapt removes adapter sequences from high-throughput sequencing reads. *EMBnet J* 2011;**17**:10–12.

3. BBMap download | SourceForge.nethttps://sourceforge.net/projects/bbmap/ (6 May 2025)

4. Patro R, Duggal G, Love MI, Irizarry RA, Kingsford C. Salmon provides fast and bias-aware quantification of transcript expression. *Nat Methods* 2017;**14**:417–419.

5. Frankish A, Diekhans M, Jungreis I, Lagarde J, Loveland JE, Mudge JM, Sisu C, Wright JC, Armstrong J, Barnes I, Berry A, Bignell A, Boix C, Sala SC, Cunningham F, Domenico T Di, Donaldson S, Fiddes IT, Girón CG, Gonzalez JM, Grego T, Hardy M, Hourlier T, Howe KL, Hunt T, Izuogu OG, Johnson R, Martin FJ, Martínez L, Mohanan S, Muir P, Navarro FCP, Parker A, Pei B, Pozo F, Riera FC, Ruffier M, Schmitt BM, Stapleton E, Suner MM, Sycheva I, Uszczynska-Ratajczak B, Wolf MY, Xu J, Yang YT, Yates A, Zerbino D, Zhang Y, Choudhary JS, Gerstein M, Guigó R, Hubbard TJP, Kellis M, Paten B, Tress ML, Flicek P. GENCODE 2021. *Nucleic Acids Res* 2021;**49**:D916–D923.

6. Dobin A, Davis CA, Schlesinger F, Drenkow J, Zaleski C, Jha S, Batut P, Chaisson M, Gingeras TR. STAR: Ultrafast universal RNA-seq aligner. *Bioinformatics* 2013;**29**:15–21.

7. Ewels P, Magnusson M, Lundin S, Käller M. MultiQC: Summarize analysis results for multiple tools and samples in a single report. *Bioinformatics* 2016;**32**:3047–3048.

8. Li X, Nair A, Wang S, Wang L. Quality control of RNA-seq experiments. *Methods in Molecular Biology* 2015;**1269**:137–146.

9. Soneson C, Love MI, Robinson MD. Differential analyses for RNA-seq: Transcript-level estimates improve gene-level inferences. *F1000Res* 2016;**4**.

10. Love MI, Huber W, Anders S. Moderated estimation of fold change and dispersion for RNA-seq data with DESeq2. *Genome Biol* 2014;**15**.

11. Zhu A, Ibrahim JG, Love MI. Heavy-Tailed prior distributions for sequence count data: Removing the noise and preserving large differences. *Bioinformatics* 2019;**35**:2084–2092.

12. Nostrand EL Van, Freese P, Pratt GA, Wang X, Wei X, Xiao R, Blue SM, Chen JY, Cody NAL, Dominguez D, Olson S, Sundararaman B, Zhan L, Bazile C, Bouvrette LPB, Bergalet J, Duff MO, Garcia KE, Gelboin-Burkhart C, Hochman M, Lambert NJ, Li H, McGurk MP, Nguyen TB, Palden T, Rabano I, Sathe S, Stanton R, Su A, Wang R, Yee BA, Zhou B, Louie AL, Aigner S, Fu XD, Lécuyer E, Burge CB, Graveley BR, Yeo GW. A large-scale binding and functional map of human RNA-binding proteins. *Nature 2020 583:7818* 2020;**583**:711–719.

13. Gu Z. Complex heatmap visualization. *iMeta* 2022;**1**:e43.

14. Pedregosa F, Varoquaux G, Gramfort A, Michel V, Thirion B, Grisel O, Blondel M, Müller A, Nothman J, Louppe G, Prettenhofer P, Weiss R, Dubourg V, Vanderplas J, Passos A, Cournapeau D, Brucher M, Perrot M, Duchesnay É. Scikit-learn: Machine Learning in Python. 2012.

15. Peterson H, Kolberg L, Raudvere U, Kuzmin I, Vilo J. gprofiler2 -- an R package for gene list functional enrichment analysis and namespace conversion toolset g: Profiler. *F1000Res* 2020;**9**.

16. Garrido-Martín D, Palumbo E, Guigó R, Breschi A. ggsashimi: Sashimi plot revised for browser- and annotation-independent splicing visualization. *PLoS Comput Biol* 2018;**14**:e1006360.

**Supplementary Figures**

**
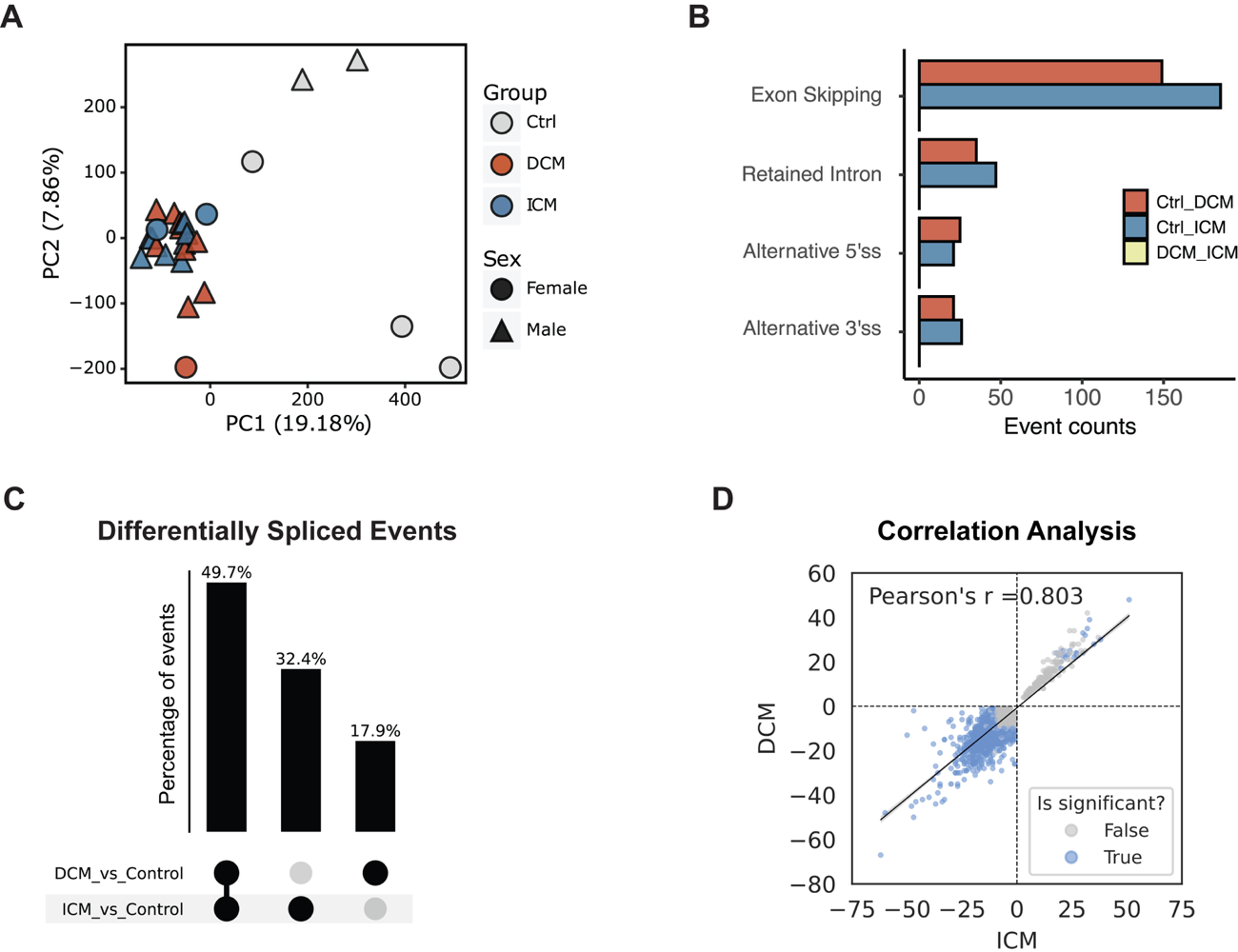
**

**Figure S1. RNA-seq analysis using *vast-tools* identifies shared splicing changes in DCM and ICM hearts.**

(A) Principal component analysis (PCA) based on PSI values demonstrates clustering of DCM and ICM splicing profiles, distinct from controls.

(B) Distribution and types of events identified as differentially spliced between controls and diseased groups (DCM and ICM).

(C) UpSet plot showing the percentage of differentially spliced events identified in pairwise comparisons: 49.7% of the differentially spliced events identified in pairwise comparisons between HF samples and controls were shared between DCM and ICM; 32.4% of events were uniquely detected in the ICM versus control comparison; 17.9% were uniquely detected in the DCM versus control comparison; and no splicing events differed between DCM and ICM hearts.

(D) Pearson’s correlation analysis of ΔPSI values between DCM and ICM relative to controls, indicating a high degree of overlap in splicing changes.

**
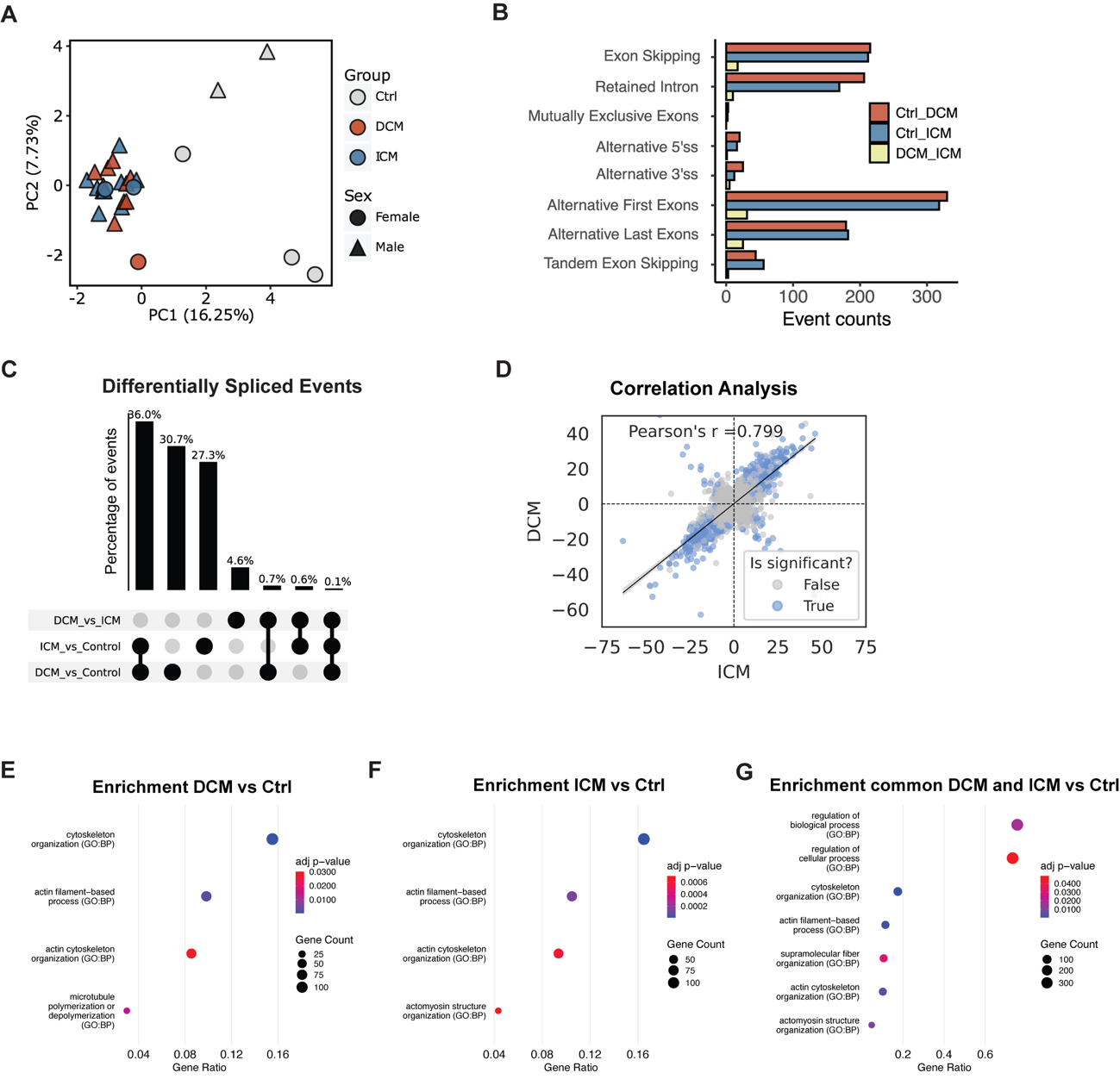
**

**Figure S2. RNA-seq analysis using *MAJIQ* identifies shared splicing changes in DCM and ICM hearts.**

(A) Principal component analysis (PCA) based on PSI values demonstrates clustering of DCM and ICM splicing profiles, distinct from controls.

(B) Distribution and types of events identified as differentially spliced between controls and heart failure patients (DCM and ICM).

(C) UpSet plot showing the percentage of differentially spliced events identified in pairwise comparisons: 36.0% of the differentially spliced events identified in pairwise comparisons between HF samples and controls were shared between DCM and ICM; 30.7% of events were uniquely detected in the ICM versus control comparison; 27.3% were uniquely detected in the DCM versus control comparison; and 4.6% of splicing events differed between DCM and ICM hearts.

(D) Pearson’s correlation analysis of ΔPSI values between DCM and ICM relative to controls, indicating a high degree of overlap in splicing changes.

(E-G) Pathway enrichment analysis of genes containing differentially spliced events identified in DCM and ICM compared to controls, as well as in the subset of events shared between both cardiomyopathy subtypes.


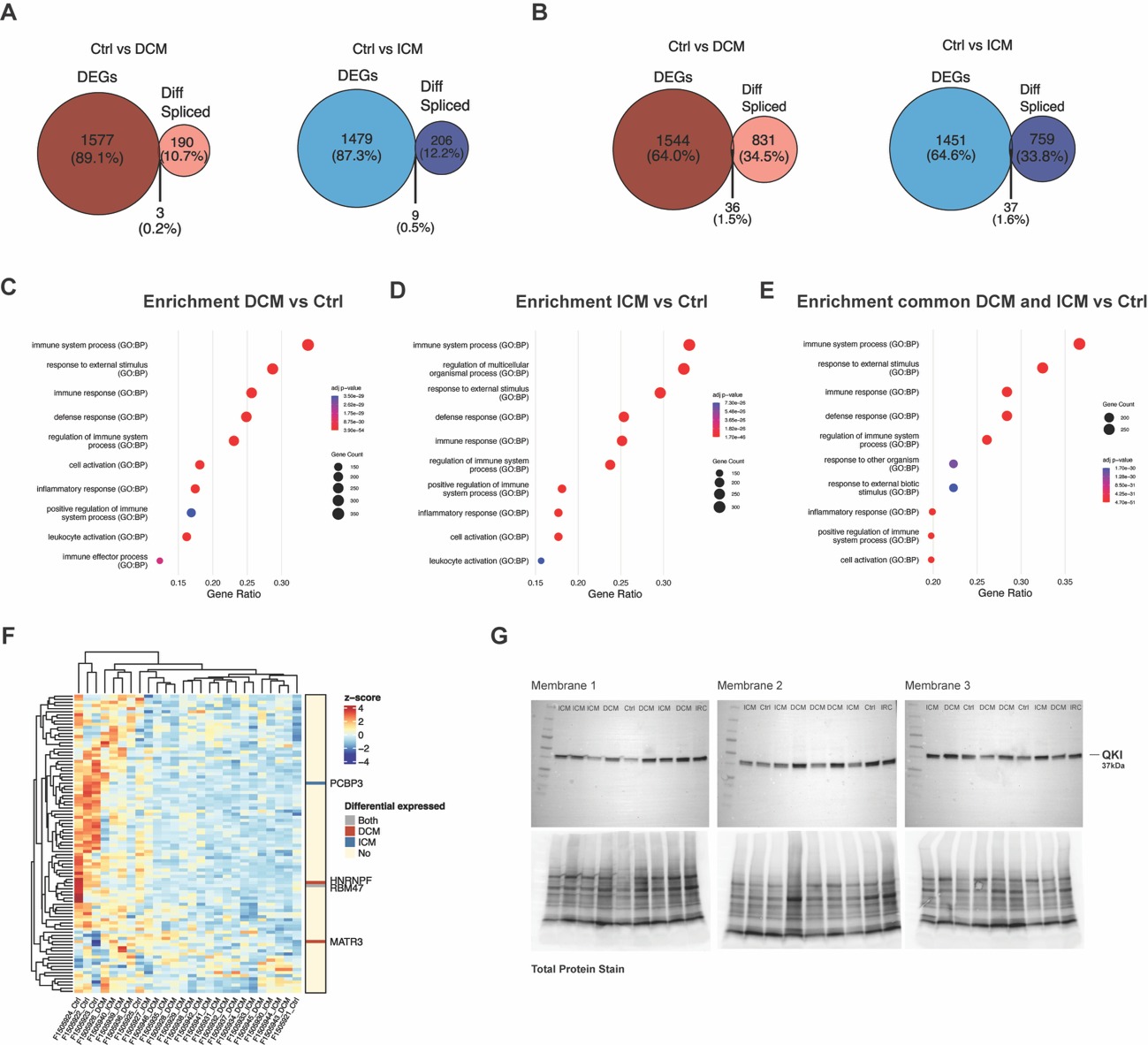


**Figure S3. Gene expression.**

(A) Venn diagram illustrating the overlap between differentially expressed genes and genes containing at least one significant splicing event, as detected by *vast-tools.*

(B) Venn diagram illustrating the overlap between differentially expressed genes and genes containing at least one significant splicing event, as detected by *MAJIQ*.

(C-E) Pathway enrichment analysis of genes differentially expressed in DCM (C) and ICM (D) compared to controls, as well as genes that were commonly differentially expressed in both disease etiologies (E).

(F) Heat map representing gene expression patterns (z-scores of normalized expression values) of a set of genes encoding RNA-binding proteins associated with splicing regulation (from ENCODE). Column clustering was performed within each group. Differentially expressed genes common to both DCM and ICM are shown in grey, those specific to DCM in red, those specific to ICM in blue, and non-differentially expressed genes in yellow, logFC=1 and p-value <0.05.

(G) Full Western Blot images of QKI protein levels in human heart tissue. Total protein staining is shown below.

**
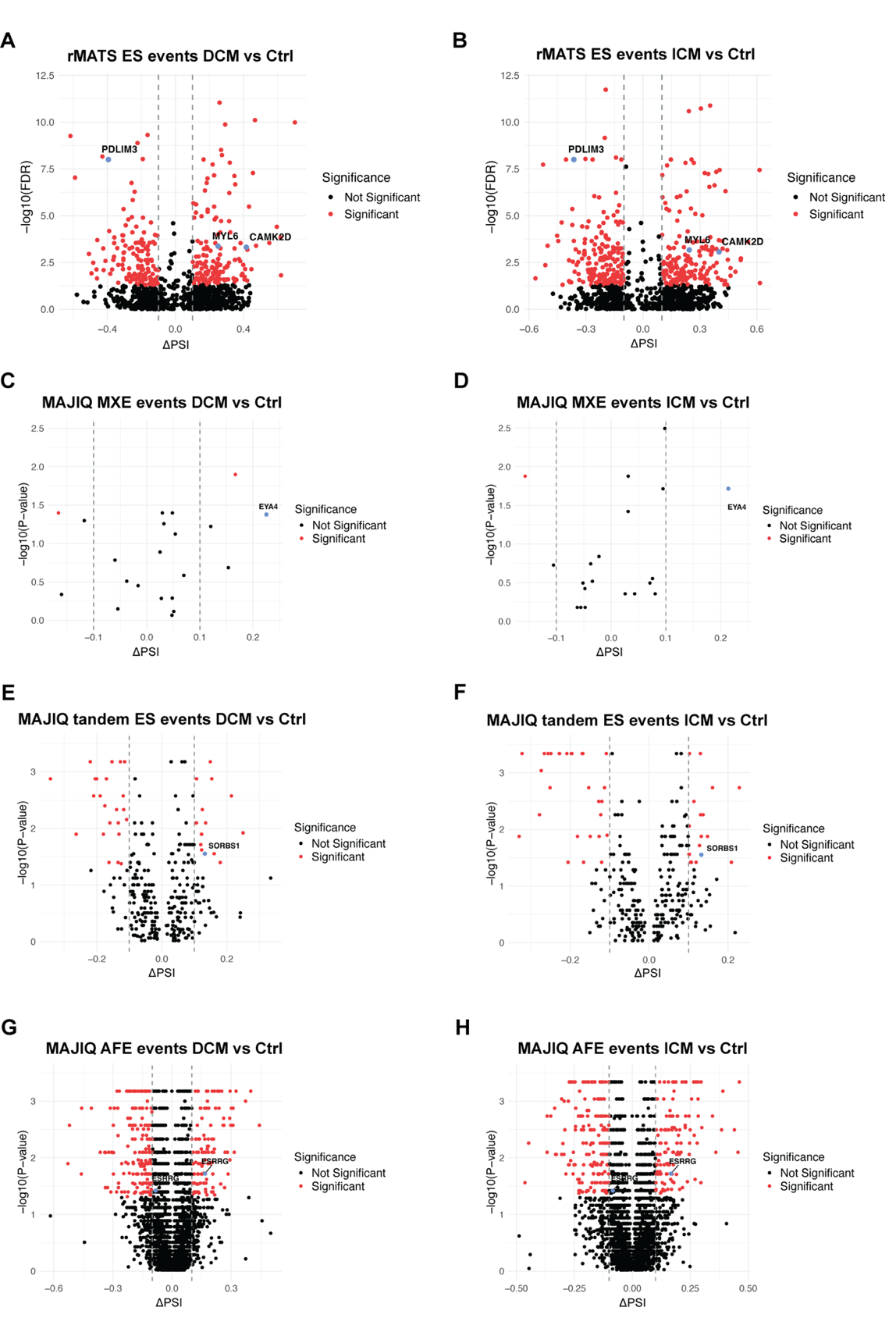
Figure S4. Prioritization of splicing events differentially regulated in both DCM and ICM samples.** Volcano plots showing splicing events detected in the comparisons DCM vs Ctrl and ICM vs Ctrl. For *rMATS*, events with FDR <0.05 ΔPSI| ≥ 0.1 are highlighted in red and considered significant. For *MAJIQ*, significant events were determined based on an absolute dPSI ≥ 0.1 and a Mann-Whitney U p-value ≤ 0.05. Events selected for experimental validation, detected in both comparisons, are highlighted in blue. Panels show: (A, B) exon skipping events identified using *rMATS*, (C, D) mutually exclusive exons (MXE) events detected using *MAJIQ*, (E, F) complex tandem exon skipping events reported by *MAJIQ* and (G, H) alternative first exons detected with MAJIQ.

**
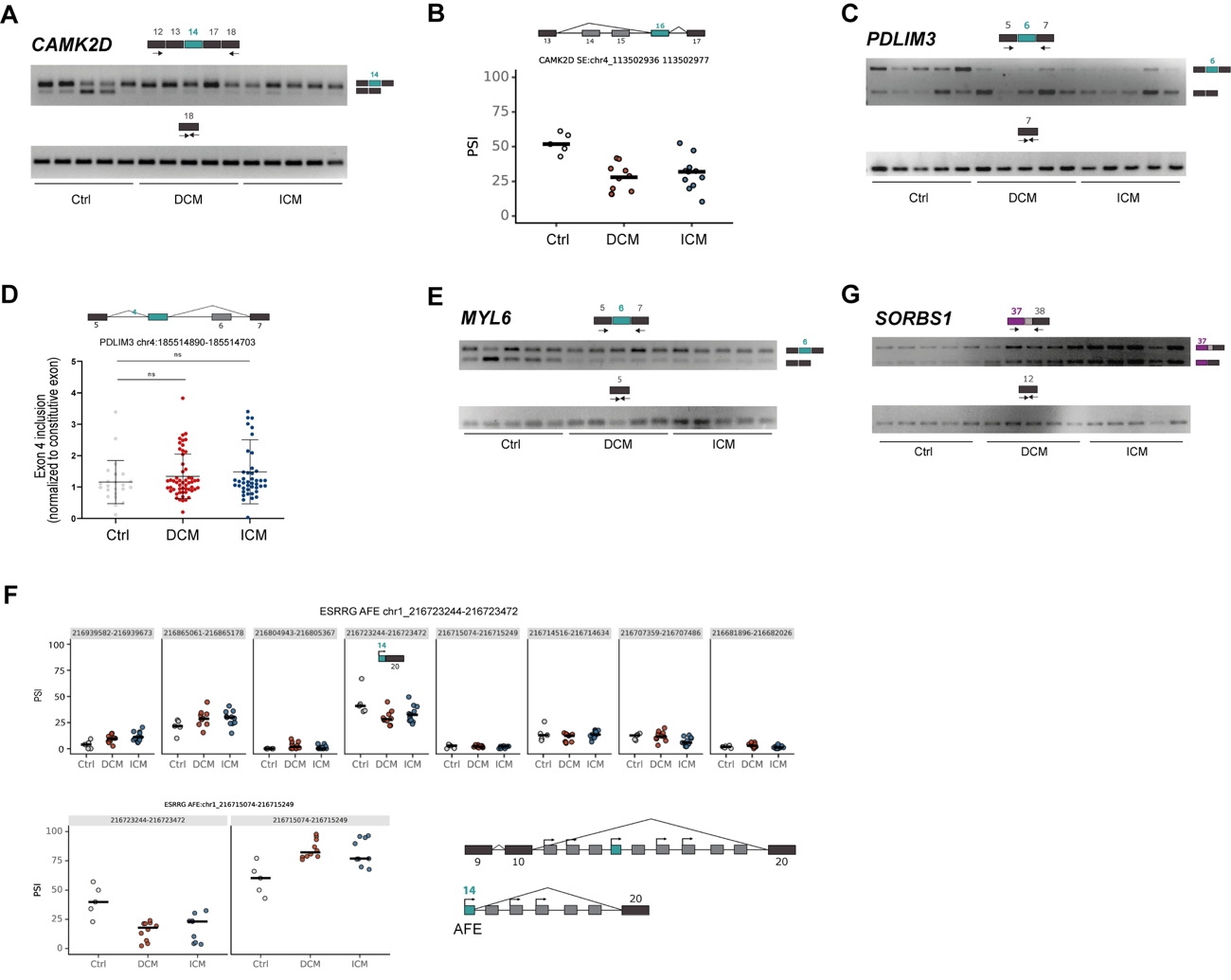
**

**Figure S5. Validation of dysregulated splicing events in *CAMK2D*, *PDLIM3*, *MYL6***, ***SORBS1* and** ***ESRRG* mRNAs.** For each mRNA, the dysregulated alternative exon is highlighted. (A, C, E, G) RT-PCR gels with primers flanking the alternative splicing event for the indicated genes. (B) PSI values from RNA-seq data for *CAMK2D* exon 16. (D) qRT-PCR quantification of *PDLIM3* exon 4 inclusion across the entire cohort, with statistical significance indicated as *ns* (*p* > 0.05). (F) For *ESRRG*, PSI values are represented for the multiple alternative first exons detected by *MAJIQ*. Arrows denote the transcription start site (AFE, alternative first exon).


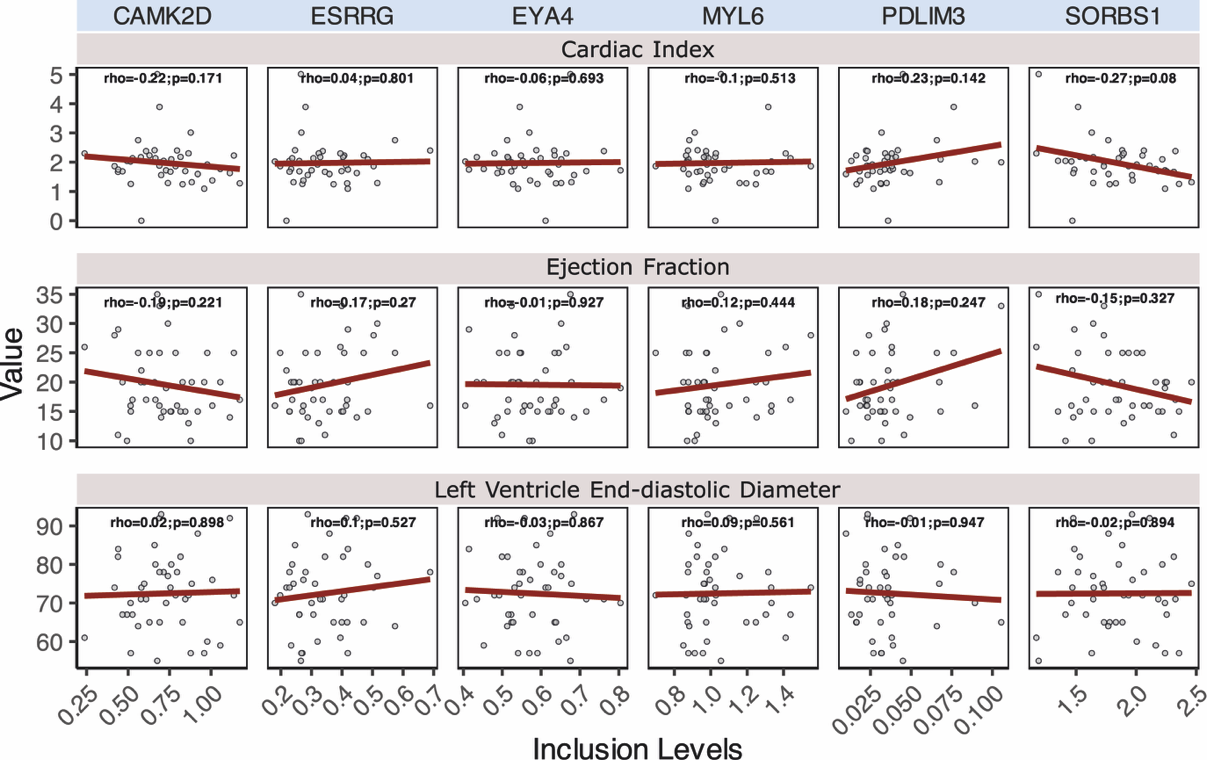


**Figure S6. Lack of correlation between splicing alterations and clinical parameters of cardiac function.**

Spearman's correlation analysis evaluating the potential association between exon inclusion levels, assessed by qRT-PCR for the indicated mRNAs, and key cardiac parameters (cardiac index, ejection fraction, and left ventricular end-diastolic diameter).

**Supplementary Tables**

**Table S1.** Demographics, clinical features (A) and metadata of the study cohort (B).

**Table S2.** Primer sequences used for RT-PCR (A) and qRT-PCR (B).

**Table S3.** Differentially spliced events identified across pairwise comparisons between DCM, ICM, and control groups using three tools (*rMATS*, *MAJIQ*, and *vast-tools*).

**Table S4.**  Overlap of alternative splicing events identified using the three tools (*rMATS*, *MAJIQ*, and *vast-tools*).

**Table S5.** Differentially expressed genes in DCM and ICM samples compared to controls.

**Table S6.** Gene expression levels of RNA-binding proteins in DCM and ICM samples compared to controls.

**Table S7.** RNA-binding protein motifs enrichment analysis using *rMAPS2*.

**Table S8.** List of the alternative splicing events identified matching an external dataset.

**Table S9.** List of RNA-binding proteins predicted to regulate the alternative splicing events validated by *cat*RAPID *omics v2.0*.

**Table S1.** Demographics (A), clinical features and metadata of the study cohort (B).

**Table S1A**

| **RNA sequencing cohort** | **Ctrl** | **DCM** | **ICM** |
| --- | --- | --- | --- |
| **Median age** | 24 | 47,5 | 59 |
| **Sex (M)** | 60% | 90% | 81,80% |
| **Cause of heart transplant** | n=3 subarachnoid haemorrhage n=2 head injury | n=10 HF | n=11 HF |
| **Extended validation cohort** | **Ctrl** | **DCM** | **ICM** |
| **Median age** | 41,5 | 49 | 62 |
| **Sex (M)** | 65% (n=15/23) | 78% (n=42/54) | 86% (n=39/45) |
| **Cause of heart transplant** | n=15 subarachnoid haemorrhage  n=8 head injury | n=54 HF | n= 45 HF |
|  |  |  |  |
|  |  |  |  |

**Table S1B**

| **Controls (n=23)** |  |  |  |  |  |  |  |
| --- | --- | --- | --- | --- | --- | --- | --- |
| **No** | **Gender** | **Age** | **Main problem** | **Comorbidities** | **RNA-Seq** |  |  |
| 1 | F | 32 | head injury | abdomen injury | Y |  |  |
| 2 | F | 22 | head injury | NA | Y |  |  |
| 3 | F | 23 | subarachnoid haemorrhage | NA | Y |  |  |
| 4 | M | 43 | subarachnoid haemorrhage | hypertension | Y |  |  |
| 5 | M | 24 | subarachnoid haemorrhage | NA | Y |  |  |
| 6 | M | 27 | subarachnoid haemorrhage | polycystic kidney, peptic ulcer disease | N |  |  |
| 7 | M | 23 | head injury | NA | N |  |  |
| 8 | M | 42 | subarachnoid haemorrhage | NA | N |  |  |
| 9 | M | 44 | head injury | alcoholism | N |  |  |
| 10 | F | 17 | head injury | NA | N |  |  |
| 11 | M | 46 | subarachnoid haemorrhage | NA | N |  |  |
| 12 | M | 52 | subarachnoid haemorrhage | NA | N |  |  |
| 13 | F | 41 | subarachnoid haemorrhage | NA | N |  |  |
| 14 | M | 44 | subarachnoid haemorrhage | alcoholism | N |  |  |
| 15 | F | 41 | subarachnoid haemorrhage | hypertension | N |  |  |
| 16 | M | 27 | head injury | NA | N |  |  |
| 17 | M | 40 | subarachnoid haemorrhage | alcoholism | N |  |  |
| 18 | F | 52 | subarachnoid haemorrhage | NA | N |  |  |
| 19 | M | 42 | subarachnoid haemorrhage | hypertension | N |  |  |
| 20 | M | 30 | head injury | NA | N |  |  |
| 21 | M | 54 | subarachnoid haemorrhage | NA | N |  |  |
| 22 | M | 27 | head injury | NA | N |  |  |
| 23 | F | 49 | subarachnoid haemorrhage | hypertension, DM post partum | N |  |  |
|  |  |  |  |  |  |  |  |
|  |  |  |  |  |  |  |  |

| **Heart Failure (n=99)** |  |  |  |  |  |  |  |
| --- | --- | --- | --- | --- | --- | --- | --- |
| **No** | **Gender** | **Age** | **Diagnosis** | **EF** | **LVED** | **CI** | **RNA-Seq** |
| 1 | F | 53 | DCM | 35 | 55 | 5,012 | Y |
| 3 | F | 58 | ICM | 17 | 67 | 2,137 | Y |
| 5 | M | 53 | DCM | 22 | 74 | 0,000 | Y |
| 7 | M | 60 | ICM | 25 | 78 | 3,891 | Y |
| 9 | F | 39 | ICM | 16 | 70 | 2,025 | Y |
| 11 | F | 43 | DCM | 15 | 57 | 1,257 | Y |
| 15 | M | 66 | ICM | 33 | 65 | 1,997 | Y |
| 21 | M | 28 | DCM | 10 | 67 | 2,044 | Y |
| 23 | M | 60 | ICM | 15 | 82 | 2,183 | Y |
| 27 | M | 35 | DCM | 20 | 75 | NA | Y |
| 29 | M | 60 | ICM | 26 | 61 | 2,301 | Y |
| 31 | M | 26 | DCM | 15 | 78 | 2,048 | Y |
| 33 | M | 54 | DCM | 15 | 80 | 2,100 | N |
| 35 | M | 45 | DCM | 16 | 78 | 2,402 | Y |
| 37 | M | 59 | ICM | 28 | 74 | 1,865 | Y |
| 39 | M | 56 | ICM | 20 | 65 | 1,248 | Y |
| 41 | M | 67 | ICM | 29 | 84 | 1,749 | Y |
| 45 | M | 40 | ICM | 14 | 92 | 1,630 | Y |
| 47 | M | 40 | DCM | 11 | 82 | 1,671 | Y |
| 49 | M | 56 | ICM | 15 | 74 | 1,677 | Y |
| 51 | M | 50 | DCM | 25 | 60 | 1,921 | Y |
| 53 | M | 51 | DCM | 19 | 70 | 1,724 | Y |
| 69 | M | 55 | DCM | 15 | 71 | 1,696 | N |
| 71 | M | 63 | ICM | 13 | 72 | 2,304 | N |
| 73 | M | 49 | DCM | 25 | 65 | 2,234 | N |
| 75 | F | 35 | DCM | 10 | 57 | 3,013 | N |
| 77 | M | 45 | DCM | 20 | 59 | 1,769 | N |
| 79 | F | 68 | DCM | 20 | 57 | 1,095 | N |
| 81 | M | 52 | ICM | 20 | 71 | 2,176 | N |
| 85 | M | 34 | DCM | 17 | 80 | 2,138 | N |
| 87 | M | 54 | DCM | 15 | 88 | 1,597 | N |
| 89 | M | 24 | DCM | 16 | 92 | 1,884 | N |
| 91 | M | 59 | ICM | 25 | 64 | 2,754 | N |
| 93 | M | 50 | DCM | 20 | 85 | 2,412 | N |
| 95 | M | 27 | DCM | 17 | 71 | 2,382 | N |
| 101 | M | 49 | DCM | 16 | 76 | 1,379 | N |
| 103 | M | 58 | ICM | 25 | 72 | 1,866 | N |
| 105 | M | 56 | DCM | 20 | 75 | 1,317 | N |
| 107 | M | 54 | DCM | 20 | 67 | 1,689 | N |
| 111 | M | 64 | DCM | 25 | 72 | 2,230 | N |
| 113 | M | 57 | ICM | 30 | 77 | 1,289 | N |
| 115 | M | 62 | ICM | 17 | 65 | 1,279 | N |
| 117 | M | 53 | DCM | 14 | 93 | 1,563 | N |
| 121 | M | 59 | ICM | NA | NA | NA | N |
| 123 | F | 53 | DCM | NA | NA | NA | N |
| 125 | M | 52 | DCM | NA | NA | NA | N |
| 127 | M | 61 | ICM | NA | NA | NA | N |
| 135 | M | 28 | DCM | NA | NA | NA | N |
| 139 | M | 62 | DCM | NA | NA | NA | N |
| 141 | M | 46 | ICM | NA | NA | NA | N |
| 149 | M | 65 | ICM | NA | NA | NA | N |
| 153 | M | 21 | DCM | NA | NA | NA | N |
| 155 | M | 34 | ICM | NA | NA | NA | N |
| 159 | F | 40 | DCM | NA | NA | NA | N |
| 161 | F | 35 | DCM | NA | NA | NA | N |
| 163 | M | 23 | DCM | NA | NA | NA | N |
| 167 | F | 59 | ICM | NA | NA | NA | N |
| 177 | F | 58 | DCM | NA | NA | NA | N |
| 181 | M | 51 | DCM | NA | NA | NA | N |
| 183 | M | 48 | DCM | NA | NA | NA | N |
| 191 | M | 63 | ICM | NA | NA | NA | N |
| 193 | M | 37 | DCM | NA | NA | NA | N |
| 197 | M | 62 | DCM | NA | NA | NA | N |
| 199 | M | 15 | DCM | NA | NA | NA | N |
| 205 | F | 53 | DCM | NA | NA | NA | N |
| 207 | M | 41 | DCM | NA | NA | NA | N |
| 209 | M | 67 | ICM | NA | NA | NA | N |
| 211 | M | 51 | DCM | NA | NA | NA | N |
| 213 | M | NA | DCM | NA | NA | NA | N |
| 215 | M | NA | ICM | NA | NA | NA | N |
| 221 | M | NA | DCM | NA | NA | NA | N |
| 223 | M | NA | ICM | NA | NA | NA | N |
| 225 | M | NA | ICM | NA | NA | NA | N |
| 227 | M | NA | DCM | NA | NA | NA | N |
| 229 | M | NA | DCM | NA | NA | NA | N |
| 231 | M | NA | ICM | NA | NA | NA | N |
| 233 | M | 62 | ICM | NA | NA | NA | N |
| 237 | M | 64 | ICM | NA | NA | NA | N |
| 239 | M | 65 | ICM | NA | NA | NA | N |
| 245 | M | 58 | DCM | NA | NA | NA | N |
| 249 | M | 61 | ICM | NA | NA | NA | N |
| 255 | M | 51 | ICM | NA | NA | NA | N |
| 257 | M | 42 | DCM | NA | NA | NA | N |
| 263 | M | 54 | ICM | NA | NA | NA | N |
| 271 | M | 62 | ICM | NA | NA | NA | N |
| 273 | M | 36 | DCM | NA | NA | NA | N |
| 277 | F | 59 | ICM | NA | NA | NA | N |
| 281 | F | 64 | ICM | NA | NA | NA | N |
| 283 | F | 45 | ICM | NA | NA | NA | N |
| 285 | M | 67 | ICM | NA | NA | NA | N |
| 293 | M | 45 | DCM | NA | NA | NA | N |
| 295 | M | 67 | ICM | NA | NA | NA | N |
| 305 | M | 61 | ICM | NA | NA | NA | N |
| 307 | M | 54 | ICM | NA | NA | NA | N |
| 309 | M | 56 | ICM | NA | NA | NA | N |
| 313 | M | 63 | ICM | NA | NA | NA | N |
| 321 | F | 61 | DCM | NA | NA | NA | N |
| 323 | M | 45 | DCM | NA | NA | NA | N |
| 325 | F | 45 | DCM | NA | NA | NA | N |

**Supplementary Table 2.** Primer sequences used for RT-PCR (A) and qRT-PCR (B).

**Supplementary Table 2A**

| **Primer** | **Sequence** | **Type** | **Amplicon Size** |
| --- | --- | --- | --- |
| *CAMK2D* Ex12Fw | TGCCATCTTGACAACTATGCT | AS event | 203 bp/239 bp |
| *CAMK2D* Ex16Rv | GCTTCAAAGTCCCCATTGTT | AS event |  |
| *CAMK2D* Ex18Fw | TCCAAAAGCAATAAACCAATCC | loading | 132 bp |
| *CAMK2D* Ex18Rv | CATTGTCTTTGGCATTCCACT | loading |  |
| *PDLIM3* Ex5Fw | ATATTCGGCCCAAACCTTTC | AS event | 170 bp/ 434 bp |
| *PDLIM3* Ex7Rv | AGAGCCATCGTCCACCATT | AS event |  |
| *PDLIM3* Ex7Fw | CCGAGTCGGACGTGTACC | loading | 107 bp |
| *PDLIM3* Ex7Rv | AGAGCCATCGTCCACCATT | loading |  |
| *MYL6* Ex5Fw | TGAGAAGATGACAGAGGAAGAAG | AS event | 114 bp/ 159 bp |
| *MYL6* Ex7Rv | GAAGGTCCTCAGCCATTCAG | AS event |  |
| *MYL6* Ex5Fw | TGAGAAGATGACAGAGGAAGAAG | loading | 73 bp |
| *MYL6* Ex5Rv | ATAGTTGATACAACCATTGCTGTCC | loading |  |
| *ESRRG* Ex14Fw | TTCCCTGCACTACGAGGAAG | AS event | 194 bp |
| *ESRRG* Ex20Rv | TCTGATGGCCATTCATGGTT | AS event |  |
| *ESRRG Ex25Fw* | TTCTGCAGAGTGCTTGGATG | loading | 160 bp |
| *ESRRG Ex25Rv* | ACCAGCTGCAGGATAGCATT | loading |  |
| *EYA4* Ex25Fw | CAACTCAACTGATCCCAGCA | AS event | 115 bp |
| *EYA4* Ex26Rv | CGCTCAAAACAGCTTTCCTT | AS event |  |
| *EYA4* Ex25Fw | CAACTCAACTGATCCCAGCA | AS event | 132 bp |
| *EYA4* Ex27Rv | TTCTGCCAAACCTTTGCATT | AS event |  |
| *EYA4* Ex23Fw | TTTTGCAACTGATGGCTTCC | loading | 151 bp |
| *EYA4* Ex23Rv | TCCAACGTTGTTCTTGTAGGTG | loading |  |
| *SORBS1* Ex35Fw | ACAAGGCATCTTCCCCATCA | AS event | 148 bp |
| *SORBS1* Ex36Rv | GATGAGCTTGCTGTGACTGG | AS event |  |
| *SORBS1* Ex37Fw | TCATTGAGAGCAGGACCAGA | AS event | 100 bp/ 230 bp |
| *SORBS1* Ex38Rv | GTGAGGTTTGACTCCTGTCG | AS event |  |
| *SORBS1* Ex12Fw | TCTGACCTTCCCGACTCTAGA | loading | 133 bp |
| *SORBS1* Ex12Rv | AGGTGGTGGTGAGAAGGATG | loading |  |

**Supplementary Table 2B**

| **Primer List** | **Sequence** | **Type** | **Amplicon Size** |
| --- | --- | --- | --- |
| *CAMK2D* Ex14Fw | TCCAGTTCGAGTGTTCAGATG | AS event | 143 bp |
| *CAMK2D* Ex16Rv | GCTTCAAAGTCCCCATTGTT | AS event |  |
| *CAMK2D* Ex18Fw | TCCAAAAGCAATAAACCAATCC | loading | 132 bp |
| *CAMK2D* Ex18Rv | CATTGTCTTTGGCATTCCACT | loading |  |
| *PDLIM3* Ex3Fw | AGCCCATCCTTTCAAAATCAACT | AS event | 171 bp |
| *PDLIM3* Ex4Rv | CTGACAGAAGAAGGGGTGCT | AS event |  |
| *PDLIM3* Ex6Fw | GGGGAAACACCTTTGATGAG | AS event | 170 bp |
| *PDLIM3* Ex7Rv | AGAGCCATCGTCCACCATT | AS event |  |
| *PDLIM3* Ex7Fw | CCGAGTCGGACGTGTACC | loading | 107 bp |
| *PDLIM3* Ex7Rv | AGAGCCATCGTCCACCATT | loading |  |
| *MYL6* Ex5Fw | TGAGAAGATGACAGAGGAAGAAG | AS event | 100 bp |
| *MYL6* Ex6Rv | CGACAGGATATGCCTCACAA | AS event |  |
| *MYL6* Ex5Fw | TGAGAAGATGACAGAGGAAGAAG | loading | 73 bp |
| *MYL6* Ex5Rv | ATAGTTGATACAACCATTGCTGTCC | loading |  |
| *ESRRG* Ex14Fw | TTCCCTGCACTACGAGGAAG | AS event | 194 bp |
| *ESRRG* Ex20Rv | TCTGATGGCCATTCATGGTT | AS event |  |
| *ESRRG Ex25Fw* | TTCTGCAGAGTGCTTGGATG | loading | 160 bp |
| *ESRRG Ex25Rv* | ACCAGCTGCAGGATAGCATT | loading |  |
| *EYA4* Ex25Fw | CAACTCAACTGATCCCAGCA | AS event | 115 bp |
| *EYA4* Ex26Rv | CGCTCAAAACAGCTTTCCTT | AS event |  |
| *EYA4* Ex25Fw | CAACTCAACTGATCCCAGCA | AS event | 132 bp |
| *EYA4* Ex27Rv | TTCTGCCAAACCTTTGCATT | AS event |  |
| *EYA4* Ex23Fw | TTTTGCAACTGATGGCTTCC | loading | 151 bp |
| *EYA4* Ex23Rv | TCCAACGTTGTTCTTGTAGGTG | loading |  |
| *SORBS1* Ex35Fw | ACAAGGCATCTTCCCCATCA | AS event | 148 bp |
| *SORBS1* Ex36Rv | GATGAGCTTGCTGTGACTGG | AS event |  |
| *SORBS1* Ex12Fw | TCTGACCTTCCCGACTCTAGA | loading | 133 bp |
| *SORBS1* Ex12Rv | AGGTGGTGGTGAGAAGGATG | loading |  |
